# ATRX loss induces multiple hallmarks of the alternative lengthening of telomeres (ALT) phenotype in human glioma cell lines in a cell line-specific manner

**DOI:** 10.1371/journal.pone.0204159

**Published:** 2018-09-18

**Authors:** Jacqueline A. Brosnan-Cashman, Ming Yuan, Mindy K. Graham, Anthony J. Rizzo, Kaylar M. Myers, Christine Davis, Rebecca Zhang, David M. Esopi, Eric H. Raabe, Charles G. Eberhart, Christopher M. Heaphy, Alan K. Meeker

**Affiliations:** 1 Department of Pathology, Johns Hopkins University School of Medicine, Baltimore, MD, United States of America; 2 Department of Oncology, Johns Hopkins University School of Medicine, Baltimore, MD, United States of America; 3 Department of Pediatric Oncology, Johns Hopkins University School of Medicine, Baltimore, MD, United States of America; 4 Department of Ophthalmology, Johns Hopkins University School of Medicine, Baltimore, MD, United States of America; 5 Department of Urology, Johns Hopkins University School of Medicine, Baltimore, MD, United States of America; University of Nebraska Medical Center, UNITED STATES

## Abstract

Cancers must maintain their telomeres at lengths sufficient for cell survival. In several cancer subtypes, a recombination-like mechanism termed alternative lengthening of telomeres (ALT), is frequently used for telomere length maintenance. Cancers utilizing ALT often have lost functional ATRX, a chromatin remodeling protein, through mutation or deletion, thereby strongly implicating ATRX as an ALT suppressor. Herein, we have generated functional ATRX knockouts in four telomerase-positive, ALT-negative human glioma cell lines: MOG-G-UVW, SF188, U-251 and UW479. After loss of ATRX, two of the four cell lines (U-251 and UW479) show multiple characteristics of ALT-positive cells, including ultrabright telomeric DNA foci, ALT-associated PML bodies, and c-circles. However, telomerase activity and overall telomere length heterogeneity are unaffected after ATRX loss, regardless of cellular context. The two cell lines that showed ALT hallmarks after complete ATRX loss also did so upon ATRX depletion via shRNA-mediated knockdown. These results suggest that other genomic or epigenetic events, in addition to ATRX loss, are necessary for the induction of ALT in human cancer.

## Introduction

Telomeres consist of multiple kilobases of repeated TTAGGG sequence at the ends of chromosomes and are protected by a sequence-specific protein cap [1]. Due to the limitations of cellular replication machinery, in the absence of a telomere length maintenance mechanism, telomeres will shorten with each cell division. In proliferating normal somatic cells, one or more telomeres will ultimately shorten to a critical length, causing cells to undergo senescence or apoptosis [2–4]. Cancer cells, given their limitless proliferation potential, evolve mechanisms to abrogate these responses and maintain their telomeres above these critical lengths. While most cancer subtypes are known to activate telomerase, which directly adds telomere DNA repeats onto the ends of chromosomes, for telomere maintenance, 5–10% of all cancers utilize a telomerase-independent mechanism, termed alternative lengthening of telomeres (ALT) [5].

While rare in cancers overall, ALT is prevalent in certain cancer subtypes, including sarcomas, pancreatic neuroendocrine tumors, and gliomas [6]. It is widely thought that ALT arises through a recombination-based mechanism, rather than direct enzymatic telomere elongation [5]. Recent evidence has implicated the break-induced repair machinery in the ALT mechanism, further suggesting a DNA-damage response component to the ALT pathway [7, 8]. In addition to the lack of telomerase activity, several other key hallmarks of ALT-positive cell populations have been identified, including the presence of ultrabright telomeric DNA foci and ALT-associated promyelocytic leukemia (PML) bodies (APBs) in a subset of cells [9], generation of circular, extrachromosomal telomeric DNA species (c-circles) [10], as well as extreme overall telomere length heterogeneity [11]. Furthermore, cancers that utilize the ALT telomere maintenance mechanism typically display loss of ATRX or, more rarely, DAXX [12–16]. ATRX and DAXX function together as a chromatin remodeling complex that load the histone variant H3.3 into telomeric and other repetitive heterochromatic regions [17–20]. In addition, ATRX itself is recruited to telomeres after the detection of replication stress, where it is thought to assist in stress relief via its intrinsic helicase activity [19, 21, 22]. Therefore, the effects of ATRX loss on telomeres may be due to either improper maintenance of telomeric heterochromatin, improper resolution of replication stress at telomeres, or both [23]. Cancers with ATRX loss display large, ultrabright telomeric DNA foci that are strongly correlated with the presence of ALT [12]. Furthermore, in adult high-grade gliomas, inactivating mutations in *ATRX* are mutually exclusive with activating mutations in the *TERT* promoter, providing genetic evidence that ATRX loss contributes to telomere maintenance via the ALT phenotype in this cancer subtype [15].

The strong link between ATRX loss and ALT in clinical specimens implies that ATRX is a suppressor of the ALT mechanism. However, existing *in vitro* data do not conclusively support this notion. Most cell lines that utilize the ALT mechanism have lost ATRX [24], while forced expression of ATRX in ALT-positive, ATRX-negative cell lines diminished the presence of ALT-associated features [25, 26]; these data suggest that ATRX loss is necessary for ALT to occur. However, several prior studies have either knocked out or knocked down ATRX in telomerase-positive cell contexts, but no evidence of the ALT phenotype has resulted from these efforts [24, 25, 27–29]. These data strongly suggest that ATRX loss alone is not sufficient for ALT to occur and that there are unidentified cooperating alterations in cancer cells that allow for the development of ALT hallmarks.

In order to better understand the apparent role of ATRX as an ALT suppressor in cancer, we have generated clonal, ATRX-knockout human high-grade glioma-derived cell lines. High-grade gliomas, encompassing grade III anaplastic astrocytomas and grade IV glioblastomas, display high frequencies of ALT, with rates being higher in pediatric cases [6, 16, 30–34]–therefore, high-grade gliomas represent an ALT-competent cancer subtype. Herein, we have identified, for the first time, two cell lines, U-251 and UW479, which generate multiple ALT-associated hallmarks after ATRX loss. These new *in vitro* tools will further our understanding of the molecular mechanisms of ALT, facilitating identification of new anti-cancer therapies for patients with ALT-positive cancers, most of whom currently face dismal prognoses and no effective treatment options.

## Results

**Fig 1:**
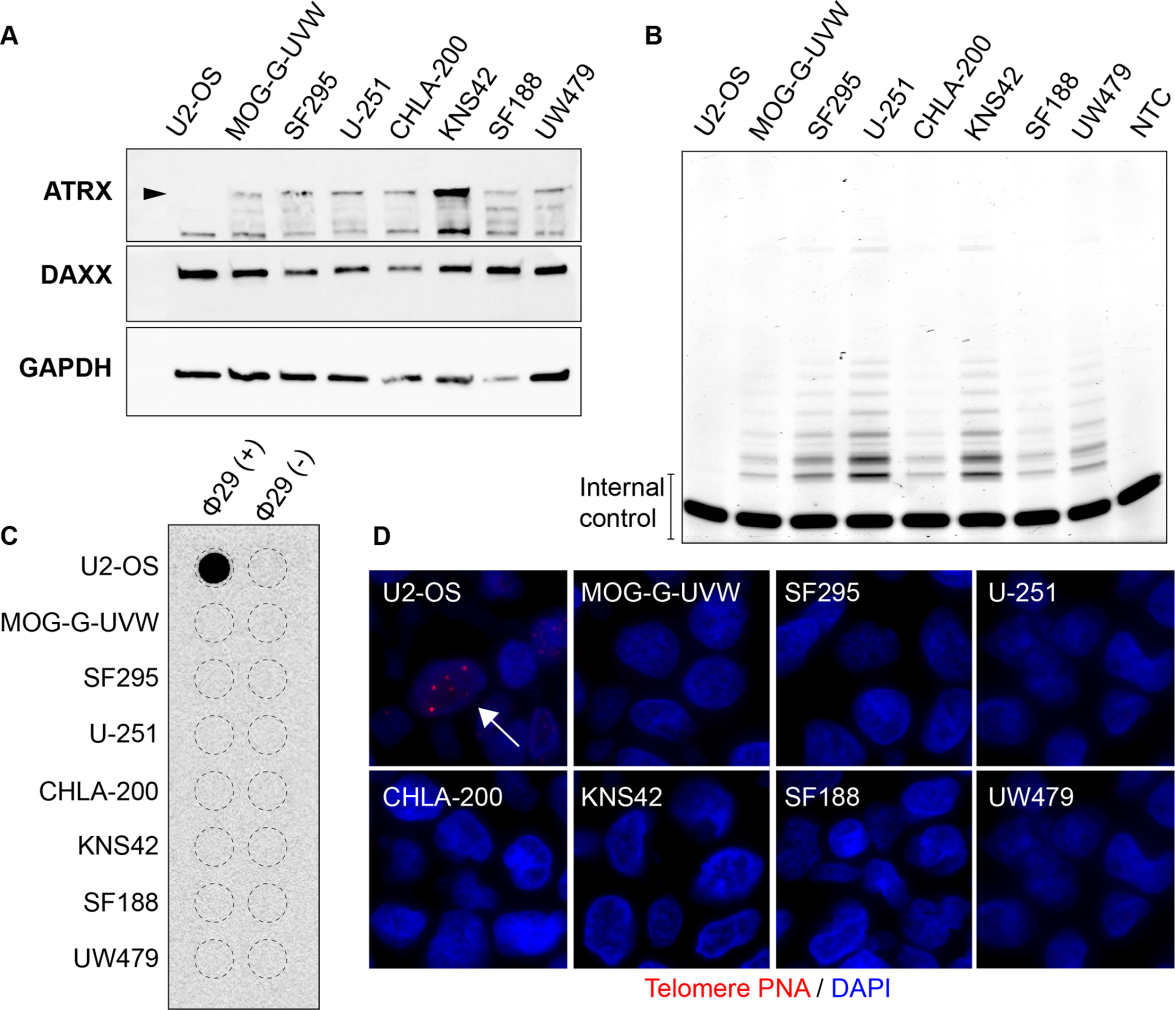
Glioma cell lines chosen for ATRX modulation. (A) Immunoblotting for known ALT suppressors ATRX and DAXX in seven glioma cell lines, as well as U2-OS, a known ALT-positive cell line with deletion of ATRX [24]. Arrowhead indicates band representing wild-type ATRX. (B) TRAP assay shows telomerase activity in all seven glioma cell lines, but not U2-OS. NTC indicates no template control. All seven glioma cell lines lack characteristics of ALT, including (C) c-circles, as measured by phi29-mediated rolling circle amplification and dot blot, and (D) ALT-associated telomere DNA foci (arrow), as assessed by telomere-specific FISH.

In order to assess the telomere-associated effects of ATRX loss, we chose a glioma *in vitro* model system, due to the relatively high frequency of ATRX loss and ALT in glioma [6, 12, 13, 16, 30–34]. To this end, we obtained a panel of high-grade glioma cell lines, derived from adult (MOG-G-UVW, SF295, and U-251) or pediatric (CHLA-200, KNS42, SF188, UW479) patients. Relevant mutations in these cell lines are listed in Table 1: activating *TERT* promoter mutations were identified in five of the seven cell lines, and previously identified mutations in *H3F3A*, encoding histone H3.3, and *TP53* were validated [35–42]. All of the glioma lines assessed retain expression of both ATRX and DAXX, as assessed by immunoblotting (Fig 1A) and immunohistochemistry (S1 Fig), while U2-OS, a well-characterized ALT-positive osteosarcoma cell line harboring an *ATRX* deletion [24, 43], lacks ATRX expression (Fig 1A). It is of note, however, that KNS42 has been reported to harbor a single nucleotide polymorphism (SNP) within its *ATRX* locus (Q891E, rs3088074) of unknown significance [44]. Furthermore, telomerase activity, measured by telomere repeat amplification protocol (TRAP), is detectable in all seven glioma cell lines and, as expected, is absent in U2-OS (Fig 1B). Conversely, all seven glioma cell lines lack characteristics of ALT activation, such as c-circles, assessed by dot blot, (Fig 1C) and ultrabright telomeric DNA foci, as detected by telomere-specific FISH (Fig 1D), both of which are apparent in U2-OS (Fig 1C and 1D). Therefore, we conclude that all seven high-grade glioma cell lines used in this study are bona fide ALT-negative, telomerase-positive cell line models.

**Table 1:** Glioma-associated mutations identified in cell lines used in this study.

	*TERT* promoter	*H3F3A*	*IDH1*	*IDH2*	*TP53*
MOG-G-UVW^A^	C228T	WT	WT	WT	WT [35–37]
SF295^A^	C228T	WT	WT	WT	R248Q [38]
U-251^A^	C228T	WT	WT	WT	R273H [38]
CHLA-200^P^	C250T	WT	WT	WT	Silent (Codon 213: CGA to CGG) [39]
KNS42^P^	C250T	G35R [40]	WT	WT	R342* [40]
SF188^P^	WT	WT	WT	WT	G266E [41]
UW479^P^	WT	WT	WT	WT	R158L [42]

A–cell line derived from adult glioma.

P–cell line derived from pediatric glioma.

WT–wild-type

*–nonsense mutation

[] indicates reference

**Fig 2:**
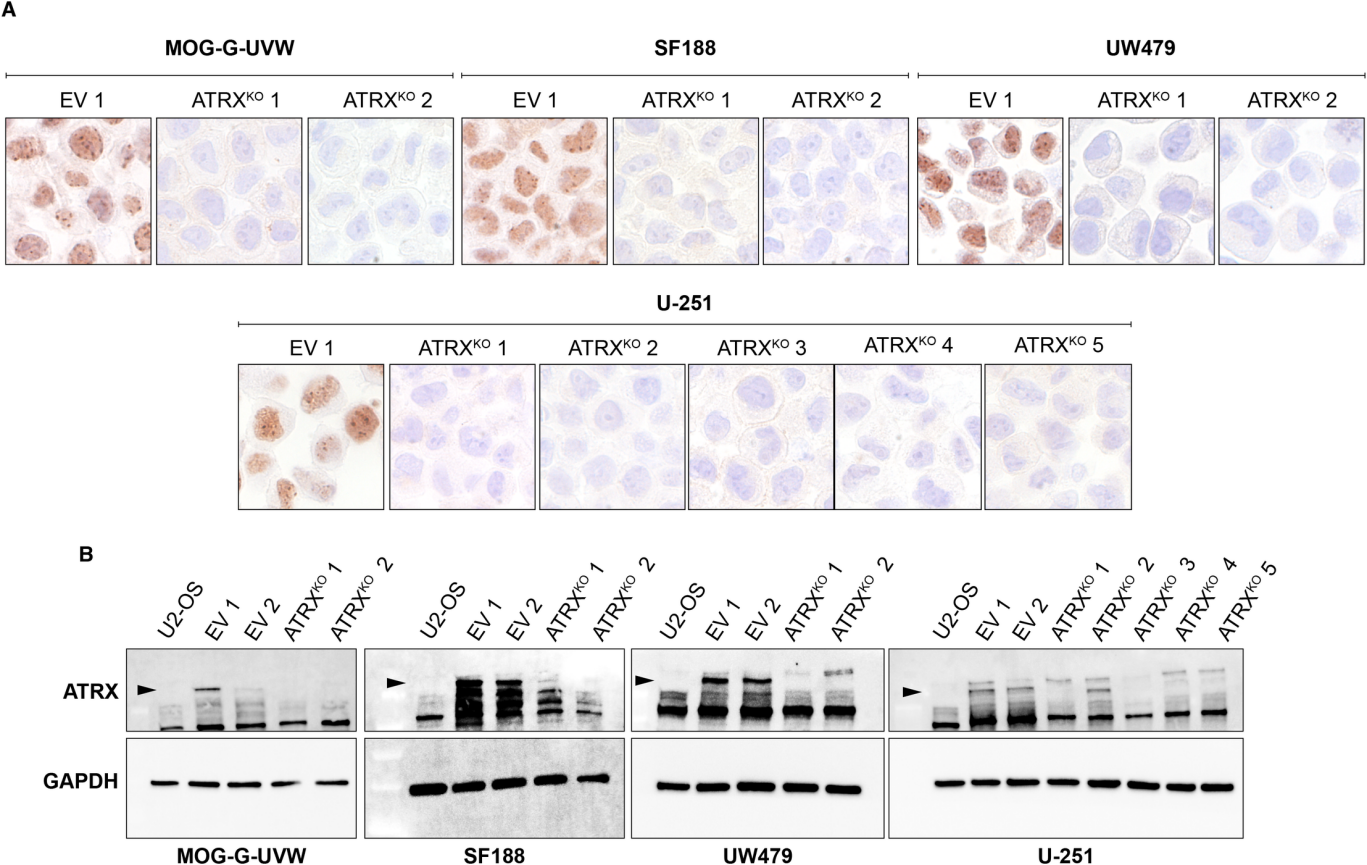
Successful elimination of ATRX expression in MOG-G-UVW, U-251, SF188, and UW479. ATRX^KO^ clones were validated by (A) immunohistochemistry and (B) immunoblotting against ATRX, compared to empty vector (EV) clones. Arrowheads indicate the band representing wild-type ATRX. For immunohistochemistry, one representative EV clone is shown. EV clones maintain ATRX protein expression and thus serve as positive controls for ATRX expression. For immunoblotting, U2-OS is included as a negative control for ATRX expression.

In order to assess the effects of ATRX loss on ALT in glioma cell lines, we sought to generate clonal cell lines with functional knockout of *ATRX* in these seven cell lines. We used a dual-nickase CRISPR approach targeting exon 9 of *ATRX* followed by the generation of ATRX^KO^ subclones, as well as clones from empty vector (EV) controls. Although knockout of ATRX was unsuccessful in SF295, CHLA-200, and KNS42, we successfully isolated at least two ATRX^KO^ clones each from MOG-G-UVW, U-251, SF188, and UW479. Immunohistochemistry of ATRX^KO^ clones reveals complete loss of nuclear immunostaining of ATRX compared to EV control clones (Fig 2A), while immunoblotting revealed a dramatic loss of ATRX, as well (Fig 2B). It is worth noting that antibodies recognizing distinct epitopes were used for the immunohistochemistry and immunoblotting experiments, indicating that the results obtained were not due to an antibody-specific effect. Both antibodies that were used recognize antigens downstream of exon 9 –the targeted region for our gene-editing approach. Therefore, we cannot exclude the possibility that a severely truncated ATRX is still present. However, all functional domains of ATRX (the ADD domain, ATP-binding helicase, and C-terminal helicase) are located downstream of the region targeted in this study. Therefore, we are confident that a severely truncated protein would be rendered completely nonfunctional.

ATRX^KO^ clones were further confirmed by Sanger sequencing of individual ATRX amplicons after colony PCR (Table 2). Nine out of eleven ATRX^KO^ clones displayed only out-of-frame sequences within the targeted region of ATRX, while the remaining two (SF188 ATRX^KO^ 1 and U-251 ATRX^KO^ 5) displayed heterozygous in-frame events that resulted in protein loss. Importantly, no wild-type ATRX sequences were observed in these clones. Taken together, these data confirm the generation of multiple, independent functional ATRX knockouts in MOG-G-UVW, SF188, U-251, and UW479.

**Table 2:** Mutations in ATRX detected in ATRX^KO^ clones.

Cell Line	Mutation
MOG-G-UVW ATRX^KO^ 1	13 bp del; 16 bp del
MOG-G-UVW ATRX^KO^ 2	58 bp del; 26 bp del; 15 bp ins+2 bp del+13 bp ins+3 bp del
U-251 ATRX^KO^ 1	16 bp del; 117 bp ins; 8 bp del+12 bp del+6 bp ins
U-251 ATRX^KO^ 2	32 bp del; 44 bp ins
U-251 ATRX^KO^ 3	20 bp del; 5 bp ins; 20 bp ins
U-251 ATRX^KO^ 4	4 bp del; 26 bp del; 35 bp del
U-251 ATRX^KO^ 5	13 bp ins; 9 bp del+42 bp ins
SF188 ATRX^KO^ 1	15 bp del; 11 bp del; 16 bp del
SF188 ATRX^KO^ 2	23 bp del; 47 bp del
UW479 ATRX^KO^ 1	14 bp del
UW479 ATRX^KO^ 2	5 bp ins; 10 bp del

**Fig 3:**
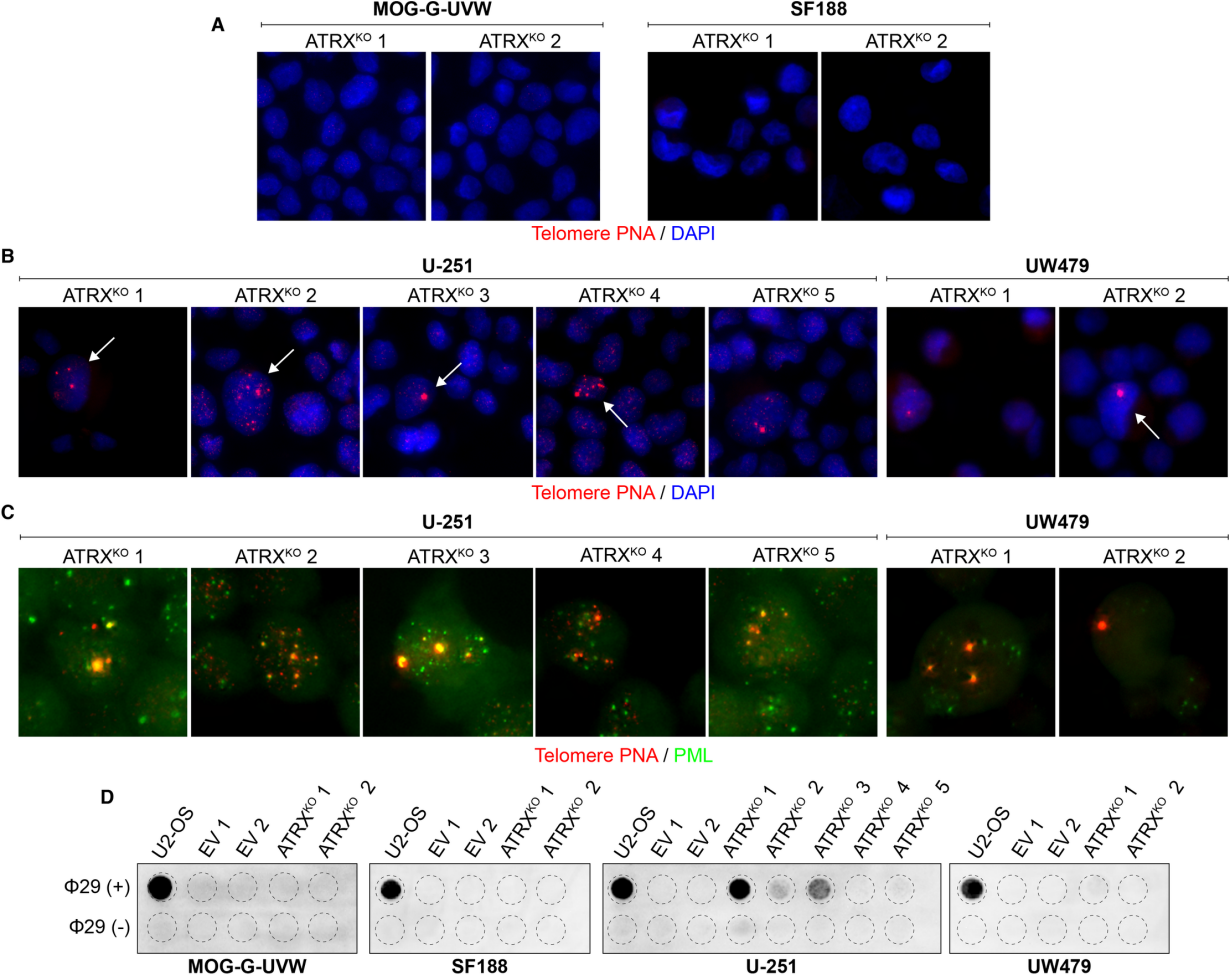
Cell line-specific induction of ALT characteristics after ATRX knockout. (A) Representative telomere FISH images indicate the absence of ultrabright telomere DNA foci in MOG-G-UVW and SF188 ATRX^KO^ clones. (B) Representative telomere FISH images indicate the presence of ultrabright telomeric DNA foci (arrows) in ATRX^KO^ U-251 and UW479 clones. (C) Colocalization of ultrabright telomere DNA foci with PML was observed in ATRX^KO^ U-251 and UW479 clones. (D) C-circles were detected in ATRX^KO^ U-251 and UW479 clones, but not ATRX^KO^ MOG-G-UVW and SF188 clones. A smaller input of U2-OS DNA (30 ng, compared to 150 ng) was included as a positive control.

Having produced functional knockouts of ATRX, we sought to determine the effect of ATRX loss on telomere maintenance in multiple clones of each of these cell lines. Specifically, we examined well-established hallmarks of the ALT telomere maintenance mechanism. Consistent with previous observations in other cell line models [24, 25, 27–29], MOG-G-UVW and SF188 ATRX^KO^ clones did not display ultrabright telomeric foci (Fig 3A). In contrast, these characteristic markers of ALT were detectable in ATRX^KO^ clones derived from U-251 and UW479 (Fig 3B), but not in empty vector control clones when assessed one month after clonal expansion (S2 Fig). In addition, each U-251 and UW479 ATRX^KO^ clone harbored ALT-associated PML bodies (Fig 3C). Furthermore, c-circles were detected in each U-251 ATRX^KO^ clone and in one out of two UW479 ATRX^KO^ clones, but not in any ATRX^KO^ clones derived from MOG-G-UVW or SF188 (Fig 3D). While the c-circle levels are quite low relative to U2-OS, samples with c-circle levels of over 4.5% of U2-OS have previously been designated c-circle positive, confirming that samples with comparatively low c-circle levels can still be considered positive [45]. Furthermore, the ALT hallmarks that we have observed were apparent even after several months in culture after clonal expansion. Therefore, while ATRX loss in MOG-G-UVW and SF188 did not lead to overt changes in telomere maintenance, loss of ATRX in U-251 and UW479 resulted in the rapid and sustained appearance of ALT-associated hallmarks.

**Fig 4:**
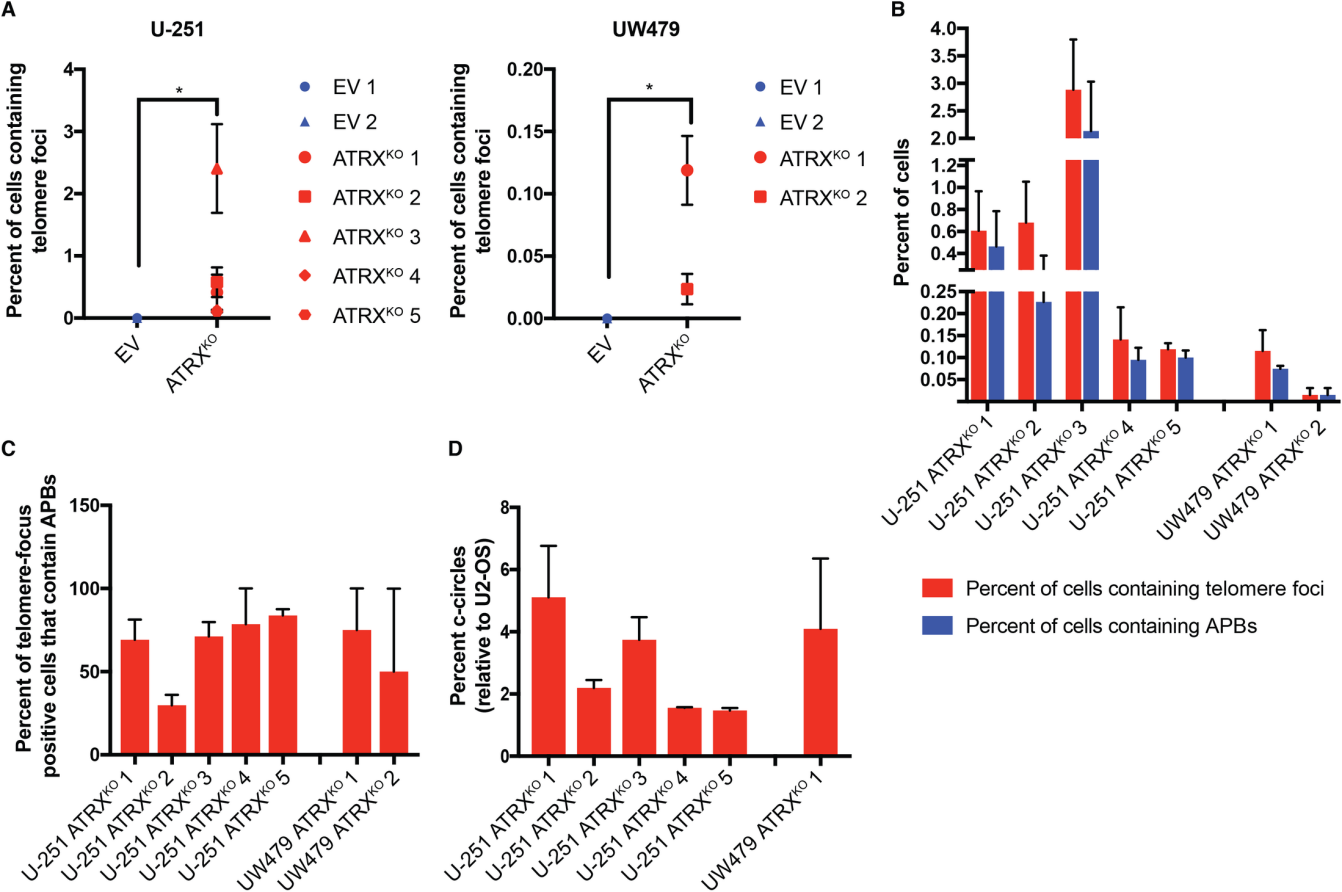
Quantification of ALT characteristics in U-251 and UW479 ATRX^KO^ clones. Telomere FISH plus DAPI nuclear staining was performed on EV and ATRX^KO^ clones, and 36–100 images (magnification = 400X) per experiment were obtained via scanning microscopy. Total cell number was determined by nuclear segmentation in TissueQuest software, and a minimum of 1000 cells were included in each analysis. (A) Cells containing ultrabright telomeric foci were identified by image analysis using pixel intensity and particle size thresholds. Analysis was limited to segmented nuclei. For both U-251 and UW479, ATRX^KO^ clones have significantly more cells containing ultrabright telomeric DNA foci than EV clones (p = 0.0004 and 0.01, respectively). Significance was calculated using Wilcoxon rank-sum analysis. Data are from three independent measurements, and error bars represent standard error of the mean. (B) Telomere FISH plus PML immunofluorescence and DAPI nuclear staining was performed on EV and ATRX^KO^ clones, and 36–100 images (magnification = 400X) per experiment were obtained via scanning microscopy. Total cell number was determined by nuclear segmentation in TissueQuest software, and a minimum of 1000 cells were analyzed for each experiment. Cells containing ultrabright telomeric foci were identified by image analysis using pixel intensity and particle size, and colocalization events were identified using the Image J Colocalization plugin[46]. Analysis was limited to segmented nuclei. The percent of all cells containing ultrabright foci and APBs were calculated (B), as was the percent of total focus-positive cells containing an APB (C). Data are from two independent experiments, and error bars represent standard error of the mean. (D) C-circle levels were quantified in ATRX^KO^ cells by densitometry and compared to U2-OS. Data were generated from three independent measurements. Error bars represent standard error of the mean.

The frequency of ultrabright telomere foci was quantified in each ATRX^KO^ clone, revealing a range in prevalence of 0.1–2.4% of cells containing these foci in the U-251 clones and 0.02–0.12% in the UW479 clones (Fig 4A). While these levels are quite low, no cells containing ultrabright telomeric foci were identified in empty vector clones–ultrabright telomeric DNA foci were significantly increased in ATRX^KO^ cells compared to empty vector clones for both U-251 (p = 0.0004) and UW479 (p = 0.01, Wilcoxon rank-sum analysis). The degree to which these foci colocalized with PML to form APBs was also quantified. A subset of ultrabright telomeric DNA foci colocalized with PML to form APBs in each ATRX^KO^ clone (Fig 4B), with 29–84 percent of focus-positive cells also containing APBs (Fig 4C). Heterogeneity in PML and telomere focus colocalization was observed both between cells and within single cells, as observed previously [12]. Furthermore, the levels of c-circles in these clones were quantified relative to a standard curve derived from U2-OS using densitometry analysis. Here, again, the levels of c-circles were low, ranging from 1–5% of the levels found in U2-OS (Fig 4D). Despite the low levels of these hallmarks that were observed in our ATRX^KO^ clones, neither c-circles nor ultrabright telomeric foci were observed in the parental cell lines (Fig 1C and 1D) or empty vector control clones (S1 Fig, Fig 3D), strongly suggesting that the low levels of ALT-associated hallmarks that are observed in these lines are, indeed, due to ATRX loss.

Another hallmark of ALT is overall telomere length heterogeneity. Therefore, we assessed the effect of ATRX loss on this ALT characteristic in ATRX^KO^ clones derived from U-251 and UW479, as well. Despite the emergence of other hallmarks of ALT in these ATRX^KO^ clones, overall telomere length heterogeneity remained largely unchanged, apart from subtle changes likely due to clonal variability, when assessed by TRF Southern blot analysis (Part A of S3 Fig) or quantitative telomere FISH (Part B of S3 Fig).

**Fig 5:**
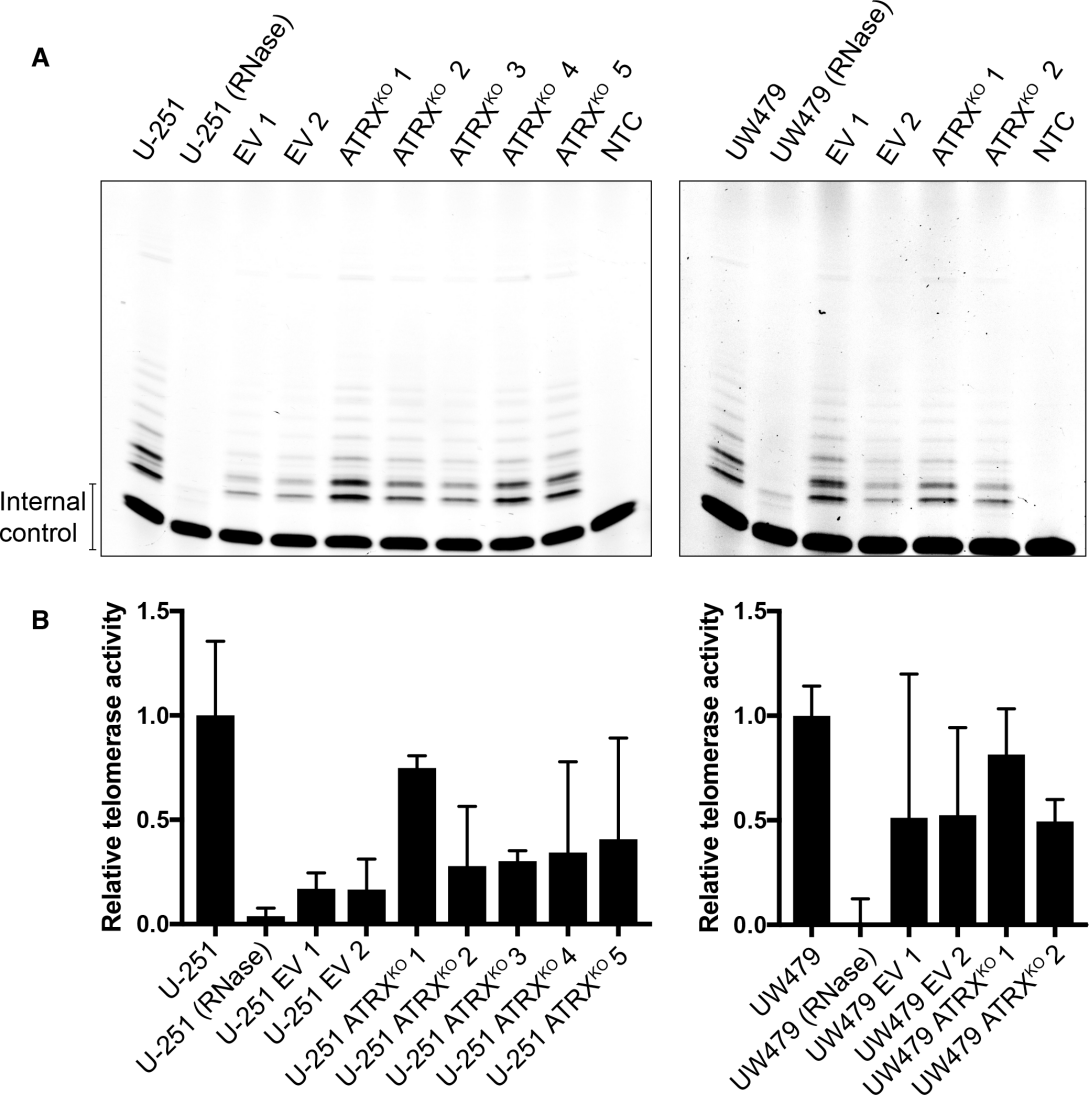
Retention of telomerase activity in ATRX^KO^ clones that display ALT-associated hallmarks. (A) TRAP analysis shows retention of telomerase activity in all EV and ATRX^KO^ clones from U-251 and UW479. NTC indicates no template control. Parental U-251 and UW479 lysates were RNase treated as specificity controls. (B) Quantification of TRAP signals reveals clonal variability in telomerase activity within EV and ATRX^KO^ clones from U-251 and UW479. Mean telomerase activity levels are shown from two independent experiments, and error bars represent standard error of the mean.

As shown in Fig 1B, both U-251 and UW479 are telomerase-positive glioma cell lines. However, hallmarks of ALT are induced after loss of ATRX in these lines (Fig 3B and 3D). Therefore, we sought to determine the potential effect of ATRX loss on telomerase activity in these clones. As shown in Fig 5, while there are slight changes in telomerase activity across the clones (presumably due to clonal variability), all ATRX^KO^ clones retain telomerase activity. Therefore, the emergence of ALT characteristics in U-251 and UW479 ATRX loss is not due to a global loss of telomerase activity; rather, ATRX^KO^ clones derived from these cell lines appear to be utilizing the telomerase enzyme to maintain telomere length, while also generating features associated with the ALT telomere maintenance mechanism.

In adult glioma, tumors with mutation of *ATRX* also tend to have mutation of *TP53* [15]. Both U-251 and UW479 harbor mutations in *TP53* (Table 1). While SF188 does as well, it is intriguing that MOG-G-UVW, derived from an adult glioma, is *TP53*-wildtype (Table 1). One interpretation of these data is that mutation of *TP53* cooperates with ATRX loss in the development of ALT in this cancer subtype. To assess whether p53 alterations cooperate with ATRX loss in promoting ALT in adult glioma, we overexpressed a dominant-negative p53 (TP53^R273H^) in MOG-G-UVW ATRX^KO^ clones (Part A of S4 Fig). ALT-associated hallmarks were assessed immediately after selection of stably transfected cells, at which point, ATRX^KO^ clones expressing TP53^R273H^ still did not display ALT-associated ultrabright telomeric DNA foci (Part B of S4 Fig) or c-circles (Part C of S4 Fig). Therefore, p53 status cannot explain the differences that we have observed regarding whether an adult glioma cell line will display ALT hallmarks after ATRX loss. In addition, recent work has suggested that downregulation of RAP1 and XRCC1 licenses *IDH1*-mutant glioma cells to engage the ALT mechanism after ATRX loss [47]. As such, we assessed the levels of RAP1 and XRCC1 in our EV and ATRX^KO^ clones by immunoblotting. Differences that were observed in RAP1 or XRCC1 levels did not correspond to the presence or absence of ALT hallmarks (S5 Fig), suggesting that these proteins are not downregulated to lead to ALT characteristics after ATRX loss in U-251 and UW479, which are *IDH1*-wildtype. However, the possibility remains that focal losses of these proteins contribute to the development of ALT characteristics in our ATRX^KO^ clones.

Furthermore, we sought to evaluate the emergence, if any, of telomere-specific DNA damage after ATRX loss. As such, we assessed the levels of telomere-dysfunction induced foci (TIFs) in our EV and ATRX^KO^ clones by assessing colocalization between telomeres and phospho-H2A.X (S6 Fig). When compared to EV clones, significant increases in TIF frequency were observed in MOG-G-UVW ATRX^KO^ 1 (p < 0.0001 compared to both EV 1 and EV 2), SF188 ATRX^KO^ 1 (p = 0.04 compared to EV 1), U-251 ATRX^KO^ 1, 2, and 3 (p < 0.0001 compared to both EV 1 and EV 2), and UW479 ATRX^KO^ 1 (p < 0.0001 compared to EV 1). Interestingly, significant decreases in TIF frequency were also observed in some clones: SF188 ATRX^KO^ 2 (p < 0.0001 compared to both EV 1 and EV 2) and UW479 ATRX^KO^ 2 (p < 0.0001 compared to EV 2). These results, as well as the significant difference observed between UW479 EV 1 and EV 2 (p < 0.0001), strongly suggest that the degree of telomere-specific damage observed in these clones is a result of clonal variability and not ATRX loss.

In addition, previous work has identified break-induced repair as a potential mechanism for ALT [7, 8]. POLD3, a component of DNA polymerase delta, was found to be necessary for break-induced telomere synthesis in ALT-positive cell lines and was found to localize to sites of induced telomeric damage [7]. In order to determine if ATRX loss affects the degree of POLD3 colocalization with telomeres, we performed simultaneous telomere FISH and immunofluorescence against POLD3 (S6 Fig). We observed a speckled, pan-nuclear localization of POLD3 rather than discrete nuclear foci in both our EV and ATRX^KO^ populations (Part A of S7 Fig). This staining pattern makes it difficult to evaluate colocalization between POLD3 signals and individual telomeres; however, the consistent staining pattern observed across our EV and ATRX^KO^ clones indicates that POLD3 does not undergo a dramatic re-localization to telomeres after ATRX loss. In addition, we did not observe a consistent colocalization pattern with ALT-associated telomeric DNA foci in ATRX^KO^ clones (Part B of S7 Fig).

**Fig 6:**
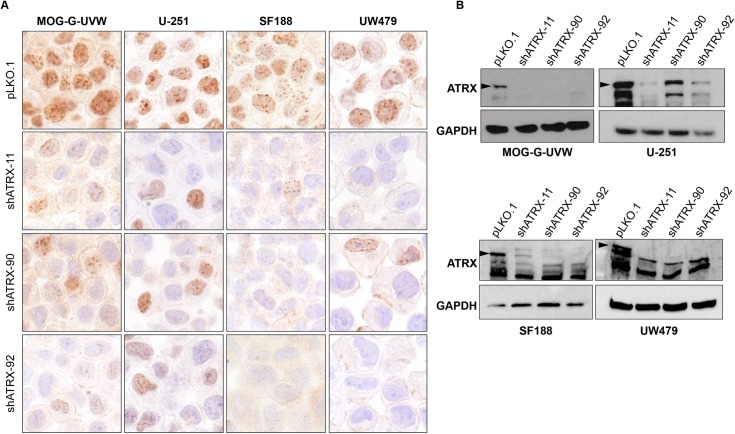
Reduction in ATRX expression using shRNAs against ATRX. ATRX knockdown using 3 different anti-ATRX shRNA lentiviral constructs in MOG-G-UVW, U-251, SF188, and UW479 was confirmed using (A) immunohistochemistry and (B) immunoblotting against ATRX. Arrowheads indicate the band representing full-length, wild-type ATRX. Empty lentiviral vector (pLKO.1) serves as a non-knockdown control for ATRX expression.

**Fig 7:**
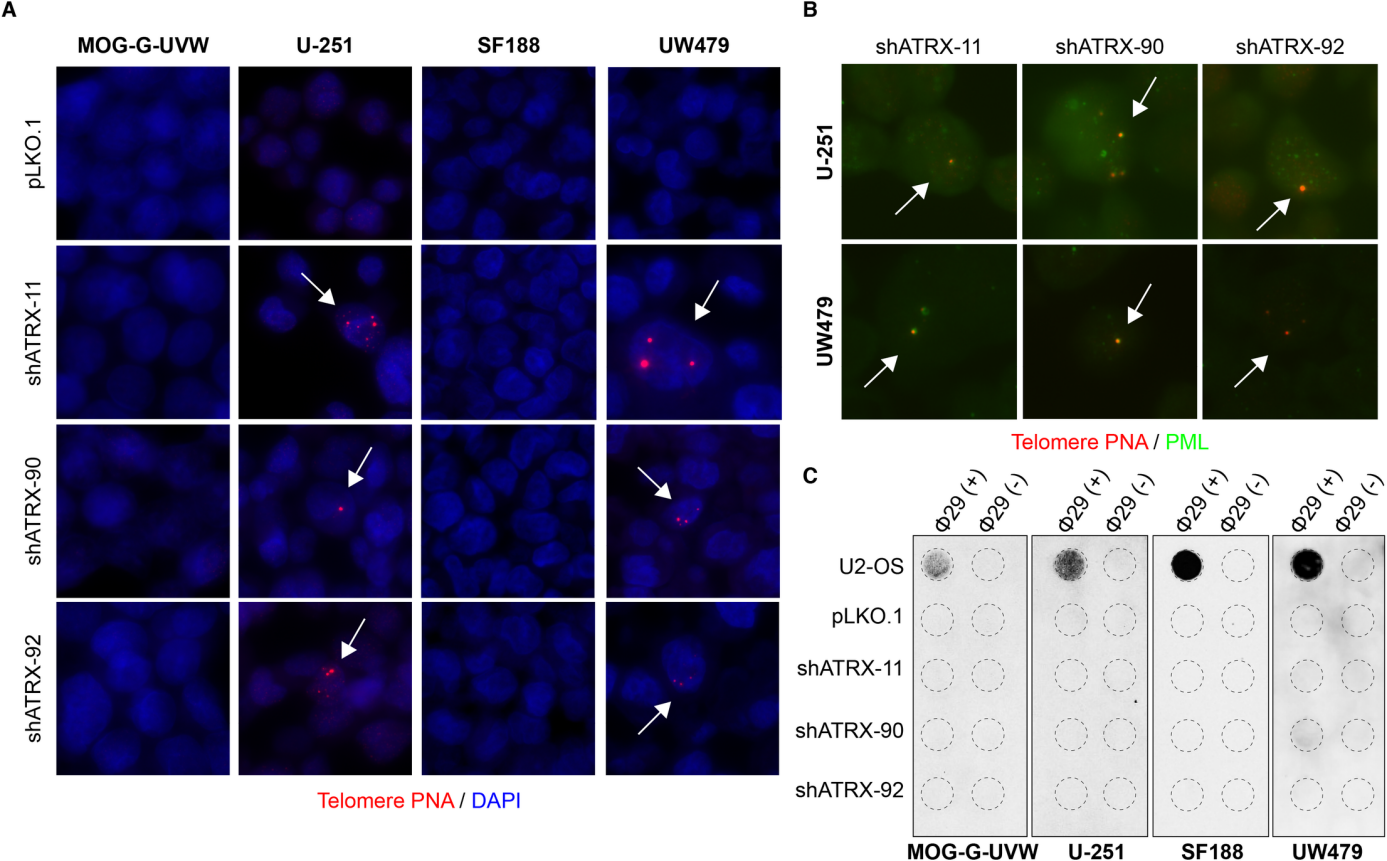
ATRX knockdown induces ALT characteristics in U-251 and UW479. (A) Representative telomere FISH images display ultrabright telomere DNA foci after ATRX knockdown in U-251 and UW479 (arrows), but not MOG-G-UVW and SF188. (B) A subset of telomeric foci that arise after ATRX knockdown colocalize with PML (arrows). (C) One out of three ATRX knockdown cells derived from UW479 (shATRX-90) shows c-circle positivity, while no other line with ATRX reduction consistently shows this feature. A lower input of U2-OS DNA (30 ng, compared to 150 ng) was included as a positive control.

In light of our observation that U-251 and UW479 develop ALT hallmarks after ATRX knockout, we sought to determine the effect of ATRX dosage on the behavior of these cell lines: in contrast to ATRX elimination, is reduction in ATRX levels sufficient to induce ALT characteristics in these cell lines? To answer this question, we stably knocked down ATRX expression in these two cell lines, as well as MOG-G-UVW and SF188, using 3 unique shRNAs against ATRX. Reduction in ATRX protein levels was confirmed by immunohistochemistry (Fig 6A) and immunoblotting (Fig 6B) compared to empty vector control (pLKO.1). ALT-associated hallmarks were assessed after selection and expansion of stable shATRX cell lines. Knockdown of ATRX yielded similar results to completely knocking out ATRX: immediately after ATRX knockdown and selection, U-251 and UW479 cells displayed ultrabright telomeric foci (Fig 7A) with PML colocalization (Fig 7B), while MOG-G-UVW and SF188 did not display this feature. Emergence of c-circles in U-251 and UW479 after ATRX knockdown was also observed but was more variable compared to ATRX^KO^. ATRX knockdown did not yield detectable c-circles in U-251, and induced positivity in one out of three shATRX lines from UW479 (Fig 7C). As expected, MOG-G-UVW and SF188 did not display ultrabright telomeric foci (Fig 7A) or c-circles (Fig 7C). Interestingly, for U-251, while the ALT-associated ultrabright foci were immediately apparent, this feature was not stable over time, in contrast to the ATRX^KO^ cells: U-251 shATRX-11 had lost ultrabright telomeric foci within ten passages (S8 Fig), while retaining reduction in ATRX levels. Still, the immediate presence of ultrabright telomere DNA foci and APBs in all three shRNA populations suggests that simply reducing the levels of ATRX is sufficient to transiently yield a subset of ALT characteristics under the appropriate conditions. Furthermore, these results indicate that the ALT features observed in U-251 and UW479 after ATRX knockout were not the result of off-target effects of the CRISPR system and can be attributed to ATRX loss.

In an attempt to identify additional glioma cell lines that show ALT hallmarks after ATRX loss, we stably knocked down ATRX in SF295, CHLA-200, and KNS42, cell lines in which we were unable to recover CRISPR-mediated ATRX knockouts. Knockdown was confirmed by immunohistochemistry (Part A of S9 Fig) and immunoblotting (Part B of S9 Fig). Similar to MOG-G-UVW and SF188, ATRX knockdown in these three lines did not yield ultrabright telomeric DNA foci (Part A of S10 Fig) or c-circles (Part B of S10 Fig).

Therefore, through our modulation of ATRX expression in glioma cell lines, we have confirmed that most cell lines do not develop ALT characteristics after ATRX loss. However, herein, we have identified for the first time two cell lines, U-251 and UW479, in which ALT hallmarks arise after partial or complete ATRX loss. These two cell lines are intriguing models for the future study of ATRX loss and ALT in glioma.

## Discussion

In this study, we have examined the effects of ATRX loss on the ALT telomere maintenance mechanism in telomerase-positive human glioma cell lines. While five of the seven cell lines examined herein do not display characteristics of ALT after ATRX knockout and/or knockdown, two cell lines–U-251 and UW479 –display multiple hallmarks of ALT immediately after functional ATRX loss. Specifically, ATRX^KO^ clones derived from U-251 and UW479 acquire ultrabright telomeric DNA foci, APBs, and c-circles (Fig 3, Fig 7). These two cell lines will be valuable tools for the future study of ALT and ATRX loss in cancer.

Ours is the first study to induce hallmarks of ALT via knockout of ATRX in established human cell lines already utilizing telomerase as their telomere length maintenance system, without altering telomerase itself. Previous induction of an ALT phenotype using *in vitro* models have been in the context of telomerase loss. Napier *et al*. induced ALT after shRNA-mediated ATRX knockdown in telomerase-negative primary fibroblasts, pre-immortalization [25], while Min *et al*. induced ALT after knocking out the telomerase subunit *TERC* in telomerase-positive cell lines [48]. In addition, O’Sullivan *et al*. stimulated the emergence of ALT characteristics after siRNA-mediated depletion of both ASF1a and ASF1b, accompanied by an intrinsic loss of TERT expression and telomerase activity [23]. Because the ATRX^KO^ clones derived from U-251 and UW479 retain enzymatically active telomerase (Fig 5), we cannot claim that the ATRX^KO^ clones are ALT-positive, despite the emergence of multiple ALT-associated hallmarks (Fig 3). The original, and most stringent, definition of ALT is a functional one, relying on demonstrating telomere maintenance over significant cell division in the absence of telomerase [11]. We cannot exclude the possibility that a sub-population of cells in ATRX^KO^ clones derived from U-251 and UW479 have lost telomerase activity and that these cells are utilizing the ALT mechanism and are the source of the ALT-associated characteristics that we detect. Without a robust method to reliably detect the individual components of the telomerase holoenzyme *in situ*, along with c-circles and ultrabright telomeric DNA foci in the same cells, we cannot yet determine whether there is intracellular heterogeneity in telomerase activity associated with ALT-activity among these cells that is subtle enough to evade detection in a bulk, *in vitro* assessment. Still, our data do support the notion that a telomerase-positive cellular population can generate ALT hallmarks after ATRX loss under the appropriate cellular context. This observation is in agreement with the prior finding that ALT and telomerase activity can coexist [49].

We have observed multiple hallmarks of the ALT mechanism in ATRX^KO^ clones derived from U-251 and UW479 –ultrabright telomeric DNA foci, APBs, and c-circles (Fig 3). It should be noted that these features have been found in contexts other than ALT. For example, APBs have been observed in mouse embryonic stem cells [50]. However, evidence of direct telomeric recombination has been detected in mouse somatic cells, suggesting that mice may simultaneously utilize ALT and telomerase to lengthen their telomeres [51]; in contrast, this phenomenon has not been observed in human cells. An additional potential source of ALT hallmarks is telomere trimming–a homeostatic mechanism to prevent telomeres from overlengthening [52]. Previous work has shown the presence of APBs [53] and c-circles [54] under instances of excessive telomere elongation leading to telomere trimming. However, telomere trimming is associated with an overall lengthening of telomeres, a feature that we do not observe in our ATRX^KO^ cells (S3 Fig). Therefore, we believe that the simultaneous presence of three ALT associated features (ultrabright telomeric DNA foci, APBs, and c-circles) after the loss of a known ALT suppressor, ATRX, is more in line with an ALT-like phenotype (while not yet fully ALT due to the retention of telomerase) than these alternative explanations.

While it is clear that ATRX loss induces the formation of ALT-associated telomere DNA foci, APBs, and c-circles in U-251 and UW479, other hallmarks of the ALT phenotype–namely, telomere length heterogeneity–were not apparent (S3 Fig). This discrepancy may be due to several reasons. First, the frequency of cells containing increased telomere content associated with telomere DNA foci are quite rare in these clones (Fig 4), and the effects of these foci on overall telomere heterogeneity may be too small to measure in a bulk analysis. Second, while ultrabright telomeric foci, APBs, and c-circles arose nearly immediately after ATRX knockout (Fig 3), it is possible that the dramatic heterogeneity in telomere length typically observed in ALT-positive specimens requires a longer culture time to develop; however, knockdown of ASF1a and ASF1b in HeLa cells resulted in dramatic increases in telomere length heterogeneity within 14 days [23], so this possibility is unlikely. Lastly, the retention of telomerase activity in these cell lines (Fig 5) may be preventing an expansion in telomere length distribution. If telomerase is still directly acting to maintain the telomeres in these cells at constant lengths, there may not be the dramatic shift in telomere lengths as is typically observed in true ALT-positive cells, where telomerase activity is absent. Ectopic expression of telomerase in ALT-positive cells causes preferential extension of the shortest telomeres in the population, reducing the number of telomere ends that are potentially recognized as DNA damage [55]. Therefore, loss of telomerase activity may be necessary for a higher degree of telomere shortening to yield both critically short telomeres and excessively long telomeres that result from recombination-based lengthening of telomeres. Possibly, this process is initiated by DNA damage signaling from chromosome ends that have lost their protective telomere caps, ultimately yielding ALT-associated extreme telomere length heterogeneity.

Herein, we have also assessed whether complete ATRX loss is necessary for the induction of ALT-associated features, or if partial suppression is sufficient. While full ATRX knockout leads to ALT features in U-251 and UW479 (Fig 3), shRNA-mediated reduction in ATRX levels also induces these characteristics in these two cell lines (Fig 7). These findings confirm that the phenotypes observed in U-251 and UW479 ATRX^KO^ clones are due to ATRX loss and not an off-target effect of CRISPR; in addition, these results have direct relevance to the current use of ATRX loss as assessed by immunohistochemistry as a diagnostic biomarker in glioma [56]. Further study will help clarify the link between partial ATRX loss in gliomas with the presence of ALT in order to more effectively use ATRX as a surrogate marker for the ALT pathway for diagnostic [56], prognostic [34, 57], and, potentially, therapeutic purposes [29, 58].

Regardless of how telomerase activity contributes to the observed ALT features in U-251 and UW479 after ATRX loss, the features that arose–ultrabright telomeric DNA foci, APBs, and c-circles–were present nearly immediately after loss of ATRX, either through CRISPR-mediated knockout (Fig 3) or shRNA-mediated knockdown (Fig 7). Therefore, U-251 and UW479 are the first identified human cancer cell lines that show ALT features after *in vitro* knockout of ATRX, a well-known and clinically relevant ALT suppressor [12, 25, 26]. The rapid emergence and long-term persistence of ALT-associated hallmarks in these cell lines after ATRX loss suggests that the telomeres in these cell lines are primed to undergo an ALT-like process, and that ATRX loss immediately allowed this mechanism to activate. In other words, there must be additional genomic and/or epigenetic events that occur in ALT-positive cancers, cooperating with ATRX loss to allow the ALT mechanism to occur. For example, either additional ALT suppressors, like ATRX, must be lost, or heretofore unidentified ALT-facilitating factors must be present. U-251 and UW479 are novel tools that will enable this investigation–further study of the underlying genomic and/or epigenetic events present in these cell lines (in comparison to cell lines that do not show ALT features after ATRX loss) will allow for a greater understanding of relevant ALT activators and suppressors in cancer. The identification of U-251 and UW479, due to their unique telomere phenotype after ATRX loss, provides a key step in the elucidation of the molecular mechanism(s) of ALT and the identification of promising therapeutic targets for ALT-positive cancers [29, 58].

In cancers, ATRX loss and ALT are tightly linked, implicating ATRX as a suppressor of the ALT pathway. Despite the strong association between ATRX loss and ALT in cancer, prior studies have not observed ALT characteristics after ATRX knockout or knockdown, much like we have observed with the MOG-G-UVW and SF188 cell lines. In contrast, here, we have identified two ALT-negative glioma cell lines–U-251 and UW479 –in which ATRX loss is sufficient for the generation of ALT hallmarks *de novo*. Because these two cell lines displayed ALT characteristics after both knockdown and knockout of ATRX, it is likely that their telomeres are already primed to undergo an ALT-like process. The identification of these cell lines will allow for the comprehensive analysis of additional genomic and/or epigenetic events–in addition to ATRX loss–that are required for suppression or licensing of the ALT mechanism.

## Materials and methods

### Cell culture

U2-OS cells were purchased from the American Type Culture Collection (ATCC; Manassas, VA). U-251 and SF295 were kindly provided by Dr. Angelo DeMarzo (Johns Hopkins University School of Medicine, Baltimore, MD), who obtained the lines as part of the NCI-60 cancer cell line panel from the National Cancer Institute. MOG-G-UVW was obtained from Sigma. CHLA-200 was obtained from the Children’s Oncology Group. KNS42 was obtained from the Japan Cancer Research Resources cell bank. SF188 and UW479 were kindly provided by Dr. Chris Jones (Institute of Cancer Research, Sutton, UK). Cells were maintained in a humidified incubator kept at 37°C with 5% CO_2_. MOG-G-UVW was grown in DMEM/F-12 (ThermoFisher) supplemented with 10% FBS and 2 mM L-Glutamine (ThermoFisher), SF295 and U-251 were grown in RPMI (ThermoFisher) supplemented with 10% FBS, CHLA-200 was grown in IMDM (ThermoFisher) supplemented with 20% FBS, 4 mM L-Glutamine, and 1X Insulin-Transferrin-Selenium, and KNS42, SF188, and UW479 were grown in DMEM/F-12 (ThermoFisher) supplemented with 10% FBS. All media contained 1% Penicillin/Streptomycin, 1% Amphotericin B, 10 μg/mL Gentamicin, and 5 μg/mL Plasmocin (Invivogen).

Identities of all cell lines used in this study were confirmed by short tandem repeat (STR) profiling using the GenePrint 10 and/or the PowerPlex 18D kits (Promega). Cells were confirmed to be mycoplasma-negative using the VenorGeM assay (Millipore-Sigma).

### Western blotting

5x10^6^ asynchronously growing cells were lysed in RIPA buffer (Cell Signaling, Cat#9806) containing a cocktail of protease inhibitors (Roche). Protein concentrations were determined by bicinchronic acid protein assay (ThermoFisher). Thirty nanograms of total protein was loaded onto a 4–12% Tris-Glycine polyacrylamide gel (BioRad) and transferred onto a nitrocellulose membrane (BioRad). Membranes were incubated overnight in primary antibody diluted in 5% milk in TBST. Antibody conditions were as follows: ATRX (1:1000, Cell Signaling, Cat#14820), DAXX (1:5000, Atlas Antibodies, Cat#HPA008736), RAP1 (1:2000, Bethyl, Cat#A300-306A), XRCC1 (1:2000, Bethyl, Cat#A300-065A) and GAPDH (1:20,000–1:30,000, Cell Signaling, Cat#5174). Membranes were incubated in secondary antibody for one hour (Anti-Rabbit HRP; Cell Signaling, Cat#7074). Signal was detected using Clarity ECL (BioRad). Blots were either exposed to film or developed using a ChemiDoc Touch (BioRad).

### Telomere repeat amplification protocol (TRAP)

TRAP was performed based on the previously described protocol [59]. Briefly, 10^6^ cells were lysed in NP-40 lysis buffer. Each assay was performed using an equivalent of 2500 cells, with RNase treated cells included as a negative control. The reactions were run on 20% TBE pre-cast gel (ThermoFisher). Gels were imaged using a ChemiDoc Touch (BioRad). Signals were quantified by densitometry analysis using ImageJ and Fiji software [60, 61]. Background intensity quantified from the no template control was subtracted from the band intensity measured for telomerase activity from cell lysates. Relative telomerase activity was determined by dividing telomerase products (background adjusted) by the band intensity of the 36 bp internal standard control.

### C-circle assay

C-circles were detected based on the previously described assay [62]. Briefly, genomic DNA was extracted from asynchronously growing cells using the DNEasy Blood and Tissue Kit (Qiagen) and purified using the QiaQuick PCR Purification Kit (Qiagen). DNA from each sample was added to a rolling circle amplification reaction in the presence or absence of phi29 polymerase (New England Biolabs). Products were blotted onto a positively-charged nylon membrane (Roche) and detected using a DIG-labeled telomere probe ((CCCTAA)_4_; Integrated DNA Technologies). Signal was detected using anti-DIG Fab Fragments (Roche, Cat#11093274910) and CDP-Star (Roche). Blots were either exposed to film or developed using a ChemiDoc Touch (BioRad). For quantification of c-circle levels, a standard curve of U2-OS, an ALT-positive, c-circle positive cell line was established by creating a dilution series of input DNA (ranging from 150 ng to 0.78 ng) was amplified and then blotted on the same membrane as the samples of interest. Signals were quantified by densitometry using ImageJ and Fiji software [60, 61].

### Telomere FISH and immunofluorescence

To generate formalin-fixed, paraffin-embedded (FFPE) blocks of cell lines, cell plugs were generated by centrifuging cells onto a 2% agarose barrier in a 0.2 mL PCR tube and immersed in 10% phosphate-buffered formalin for 48 hours.

Cell plugs were subsequently paraffin-embedded and sectioned at 5 μM thickness. Alternatively, cells were fixed in 10% phosphate-buffered formalin for 30 minutes and spun directly onto slides using a Shandon Cytospin 2. Telomere-specific *in situ* hybridization (FISH) was performed as previously described [63]. Briefly, antigen retrieval was performed using citrate, pH 6 (Vector Laboratories, Cat#H-3300). Slides were then re-hydrated and exposed to a Cy3-labeled peptide nucleic acid (PNA) probe complementary to telomeric DNA (N-CCCTAACCCTAACCCTAA-C). Subsequently, slides were incubated in primary antibody against PML (1:100 for two hours; Santa Cruz, Cat#sc966), phospho-H2A.X (Ser 139) (1:200 for two hours; MilliporeSigma; Cat#05–636), or POLD3 (1:100 for one hour; Atlas Antibodies Cat#HPA058846) and for 30 minutes in secondary antibody (1:100; ThermoFisher, Cat#A21237 or A11001). Finally, slides were washed prior to DAPI counterstaining.

### Sequencing

Genomic DNA was isolated from asynchronously growing cells using the DNEasy Blood and Tissue Kit (Qiagen). Twenty nanograms of genomic DNA was amplified using GoTaq Green polymerase (Promega) using cycling conditions optimal for each primer pair (S1 Table). PCR products were purified using QIAQuick PCR Purification Kit (Qiagen) and sequenced by Eurofins Genomics.

### ATRX knockout

Guide RNAs (gRNAs) targeting ATRX were designed using the algorithms available at crispr.mit.edu [64]. gRNAs were selected that targeted ATRX exon 9 (5’-TGGACAACTCCTTTCGACCA-3’ and 5’-TAATGGATGAAAACAACCAA-3’). gRNAs were cloned into the GFP-tagged Cas9n plasmid, PX461, which was a gift from Feng Zhang (Addgene #48140) [65]. Cells were co-transfected with both gRNA-Cas9n plasmids or the empty Cas9n plasmid using Lipofectamine 3000 (ThermoFisher). Clones were generated by single cell sorting by GFP positivity into a 96-well tissue culture plate for cloning or were isolation by limiting dilution plating. Clones were screened for insertions or deletions in ATRX using RT-PCR (S1 Table). RT-PCR amplicons of interest were TOPO-TA cloned (ThermoFisher), and colony PCR was performed prior to Sanger sequencing (Eurofins Genomics).

### Immunohistochemistry

FFPE slides were deparaffinized and hydrated prior to antigen retrieval. For ATRX staining, slides were steamed in EDTA buffer (Invitrogen, Cat#5500 or AM9849) for 45 minutes, and for DAXX staining slides were steamed in citrate buffer (Vector Laboratories, Cat#H-3300) for 25 minutes. Slides were blocked twice (Dako, Cat#s S2003 and X0909), then were incubated for one hour in primary antibody against ATRX (1:500; Atlas Antibodies, Cat# HPA001906) or DAXX (1:150; Atlas Antibodies, Cat#HPA008736). Antibody signal was detected using HRP-conjugated Anti-Rabbit IgG (Leica, Cat#PV6119) and visualized by 3,3’ diamino-benzidine (Millipore-Sigma, Cat#D1468) prior to hematoxylin counterstaining (Dako, Cat#S2009), dehydration, and mounting.

### Quantification of telomere FISH and PML immunofluorescence

Combined telomere FISH and PML immunofluorescence was performed on cytospins as described above. The TissueFAXS Plus (Tissue Gnostics) automated microscopy workstation was utilized for slide-based imaging. The platform contains an 8-slide ultra-precise motorized stage for high-throughput scanning and utilizes a Zeiss Z2 Axioimager microscope with high quality optics. A separate high-performance workstation with TissueQuest software (Tissue Gnostics) was used to analyze the fluorescent images. For each cell line, automated, precise nuclear segmentation was performed to generate total cell number. Images containing the segmentation masks, along with telomere and PML signals, were exported for analysis using using ImageJ software [60]. Background subtraction was performed for PML images using a rolling ball algorithm, and a threshold was applied to the remaining spots. Rolling ball and threshold values were empirically determined for each set of images. Colocalization between telomere foci and PML bodies was identified using the Colocalization plugin for ImageJ [46]. All analyses were limited to segmented nuclei.

### TRF Southern blotting

Cells were resuspended in proteinase K lysis buffer (10 mM Tris-HCl, pH 8; 100 mM EDTA; 0.5% SDS; 20 μg/mL RNase A; 100 μg/mL proteinase K) and incubated at 37°C with agitation for five hours. Two equal volume phenol:chloroform:isoamyl alcohol (25:24:1, pH 8) extractions were performed, followed by two ethanol precipitations and final resuspension in H_2_O. DNA was digested overnight with AluI and MboI (New England Biolabs). Two micrograms of digested DNA were run on an 0.8% agarose gel for 4 hours at 100 V. The gel was depurinated, denatured, and neutralized prior to transfer onto a nylon membrane using the Turboblotter transfer system (GE). Detection was performed using a DIG-labeled telomere probe ((CCCTAA)_4_; Integrated DNA Technologies). Signal was detected using CDP-Star (Roche). Blots were either exposed to film or developed using a ChemiDoc Touch (BioRad).

### TIF analysis

Telomere-specific FISH and immunofluorescence against phospho-H2A.X was performed on formalin-fixed, paraffin-embedded cell blocks as described above. Slides were imaged using a TissueFAXS Plus (Tissue Gnostics) automated microscopy workstation, and images were obtained using TissueQuest software (Tissue Gnostics) on a separate workstation, as described above. Images containing telomere and phospho-H2A.X signals were exported for analysis using using ImageJ software [60]. For both telomeres and phospho-H2A.X signals, background subtraction was performed using a rolling ball algorithm, and an intensity threshold was applied to the remaining spots. Threshold values were empirically determined for each set of images. Finally, in order to exclude ultrabright telomeric foci and cells expressing abnormally high levels of phospho-H2A.X, only spots smaller than 20 pixels were included in the final analysis. Colocalization between telomeres and phospho-H2A.X puncta were identified using the Colocalization plugin for ImageJ [46].

### ATRX knockdown

shRNA constructs targeting ATRX (TCN000034811, TCN000034790 and TRCN0000013592), as well as pLKO.1 empty vector (SHC001), were obtained from Millipore-Sigma. Lentiviral particles for each shATRX construct and the pLKO.1 empty vector were produced by co-transfecting these plasmids with a VSVG packaging plasmid into 293T cells using Lipofectamine 2000 (ThermoFisher). Supernatants were collected at 48 and 72 hours post-transfection and concentrated with PEG8000 precipitation. Glioma cells were transduced with shATRX or empty vector viral particles for 48 hours prior to selection with puromycin.

### Dominant-negative mutant TP53 overexpression

Cells were transfected with a R273H dominant-negative mutant TP53 expression construct, which was a gift from Bert Vogelstein (Addgene #16439) [66], using Lipofectamine 3000. Stably transfected cells were selected by treatment with G418-Sulfate. RNA was extracted from cells using PerfectPure (5-Prime) and converted to cDNA using RealMasterScript Supermix (5-Prime), and p53 transcripts containing the dominant-negative mutation were detected by Sanger sequencing.

### Microscopy

Fluorescent images were obtained using a Nikon 50i epifluorescence microscope equipped with X-Cite series 120 illuminator (EXFO Photonics Solutions Inc.) and appropriate fluorescence excitation/emission filters. Grayscale images were captured using Nikon NIS-Elements software and an attached Photometrics CoolsnapEZ digital camera, pseudo-colored and merged. Alternatively, fluorescent images were obtained using a TissueFAXS Plus (Tissue Gnostics) automated microscopy workstation with a Pixelfly USB monochrome camera (PCO). Fluorescent background was subtracted using Adobe Photoshop software. Brightfield images were obtained using a Nikon 50i epifluorescence microscope, Nikon NIS-Elements software, and an attached Nikon Digital Sight DS-Fi1 camera.

### Statistical analysis

Significance was determined using Wilcoxon rank-sum analysis using the R environment [67]. P values less than 0.05 were considered significant. All graphs were generated using Graphpad Prism software (versions 7 and 8).

## Supporting information

S1 Table Sequencing specifications. PCR primer sequences and associated PCR amplification conditions.
(DOCX)

S1 Fig Immunohistochemistry of ATRX and DAXX in glioma cell lines. All glioma cell lines utilized in this study retain nuclear expression of both proteins, with formation of characteristic nuclear puncta.
(TIF)

S2 Fig Empty vector clones lack ultrabright telomeric DNA foci. Representative images of telomere FISH from EV clones isolated from MOG-G-UVW, SF188, U-251 and UW479 indicate a lack of ALT-associated ultrabright telomere DNA foci.
(TIF)

S3 Fig ATRX^KO^ clones derived from U-251 and UW479 do not display increased telomere length heterogeneity. (A) TRF Southern blot analysis does not reveal gross changes in telomere lengths between parental, empty vector, and ATRX^KO^ cells. (B) Measurement of telomere pixel intensity after quantitative telomere FISH does not reveal increased heterogeneity in overall telomere content between empty vector and ATRX^KO^ cells.
(TIF)

S4 Fig Concomitant p53 mutation and ATRX loss are not sufficient for induction of ALT characteristics. **(**A) The R273H dominant-negative variant of p53 was stably overexpressed in ATRX-knockout MOG-G-UVW cells. This mutation did not result in (B) ultrabright telomeric DNA foci or (C) c-circles. A smaller input of U2-OS DNA (30 ng, compared to 150 ng) included as a positive control.
(TIF)

S5 Fig Reduced RAP1 and XRCC1 expression are not observed in ATRX^KO^ clones displaying ALT hallmarks. RAP1 and XRCC1 levels were assessed in EV and ATRX^KO^ clones by immunoblotting. No consistent changes in expression of these proteins were observed after ATRX loss in clones showing ALT hallmarks.
(TIF)

S6 Fig Quantification of telomere-specific DNA damage after ATRX loss. Combined telomere-specific FISH and immunofluorescence against phospho-H2A.X was performed in EV and ATRX^KO^ clones, and 36 images (magnification = 400X) per experiment were obtained via scanning microscopy. A minimum of 2000 cells were analyzed for each clone. Telomeres and phospho-H2A.X puncta were identified by setting pixel intensity thresholds after background subtraction. Ultrabright telomeric foci and cells overexpressing phospho-H2A.X were excluded from analysis by eliminating signals larger than 20 pixels. Colocalization events were identified using the Image J Colocalization plugin [46], and percent colocalization was calculated as a fraction of total telomeres. Significance was calculated using a one-way ANOVA incorporating a Tukey’s multiple comparisons test. Asterisks (*) indicate significant difference from the EV1 clone, while pound signs (#) indicate significant difference from the EV2 clone. Error bars represent standard deviation.
(TIF)

S7 Fig ATRX loss does not induce POLD3 focus formation. Combined telomere-specific FISH and immunofluorescence against POLD3 was performed in EV and ATRX^KO^. A) In both EV and ATRX^KO^ clones, a pan-nuclear, speckled pattern was observed for POLD3. Representative images (magnification = 400X) for EV and ATRX^KO^ clones from MOG-G-UVW, U-251, and UW479 are shown. B) No consistent pattern of colocalization between POLD3 and ALT-associated telomeric DNA foci was observed. Representative images (magnification = 400X) of cells from U-251 ATRX^KO^ 1 are shown.
(TIF)

S8 Fig Loss of ALT-associated hallmarks in later-passage U-251 shATRX cells. Representative telomere FISH from U-251 shATRX cells indicates that, while ultrabright telomeric DNA foci persist in U-251 shATRX-90 and U-251 shATRX-92, this ALT hallmark is no longer present in U-251 shATRX-11 after over ten passages.
(TIF)

S9 Fig Confirmation of ATRX knockdown in SF295, CHLA-200, and KNS42. ATRX knockdown in SF295, CHLA-200, and KNS42 was confirmed using (A) immunohistochemistry and (B) immunoblotting against ATRX. Arrowhead indicates band representing full length wild-type ATRX.
(TIF)

S10 Fig Lack of ALT hallmarks after ATRX knockdown in SF295, CHLA-200, and KNS42. (A) Representative telomere FISH images reveal no telomeric foci formation after ATRX knockdown in SF295, CHLA-200, or KNS42. (B) ATRX knockdown does not induce c-circle formation after ATRX knockdown in SF295, CHLA-200, or KNS42. A lower input of U2-OS DNA (30 ng, compared to 150 ng) was included as a positive control.
(TIF)

## References

[1] Shay JW, Wright WE. Role of telomeres and telomerase in cancer. Semin Cancer Biol. 2011;21(6):349–53. 10.1016/j.semcancer.2011.10.001 ; PubMed Central PMCID: PMCPMC3370415. 22015685 PMC3370415

[2] Hayflick L. The limited in vitro lifetime of human diploid cell strains. Exp Cell Res. 1965;37:614–36. . 14315085 10.1016/0014-4827(65)90211-9

[3] Allsopp RC, Chang E, Kashefi-Aazam M, Rogaev EI, Piatyszek MA, Shay JW, et al Telomere shortening is associated with cell division in vitro and in vivo. Exp Cell Res. 1995;220(1):194–200. 10.1006/excr.1995.1306 . 7664836

[4] Allsopp RC, Harley CB. Evidence for a critical telomere length in senescent human fibroblasts. Exp Cell Res. 1995;219(1):130–6. 10.1006/excr.1995.1213 . 7628529

[5] Cesare AJ, Reddel RR. Alternative lengthening of telomeres: models, mechanisms and implications. Nat Rev Genet. 2010;11(5):319–30. 10.1038/nrg2763 . 20351727

[6] Heaphy CM, Subhawong AP, Hong SM, Goggins MG, Montgomery EA, Gabrielson E, et al Prevalence of the alternative lengthening of telomeres telomere maintenance mechanism in human cancer subtypes. Am J Pathol. 2011;179(4):1608–15. 10.1016/j.ajpath.2011.06.018 ; PubMed Central PMCID: PMCPMC3181356. 21888887 PMC3181356

[7] Dilley RL, Verma P, Cho NW, Winters HD, Wondisford AR, Greenberg RA. Break-induced telomere synthesis underlies alternative telomere maintenance. Nature. 2016;539(7627):54–8. Epub 2016/11/04. 10.1038/nature20099 ; PubMed Central PMCID: PMCPMC5384111. 27760120 PMC5384111

[8] Min J, Wright WE, Shay JW. Alternative Lengthening of Telomeres Mediated by Mitotic DNA Synthesis Engages Break-Induced Replication Processes. Molecular and cellular biology. 2017;37(20). Epub 2017/08/02. 10.1128/MCB.00226-17 ; PubMed Central PMCID: PMCPMC5615184. 28760773 PMC5615184

[9] Yeager TR, Neumann AA, Englezou A, Huschtscha LI, Noble JR, Reddel RR. Telomerase-negative immortalized human cells contain a novel type of promyelocytic leukemia (PML) body. Cancer Res. 1999;59(17):4175–9. Epub 1999/09/15. . 10485449

[10] Cesare AJ, Griffith JD. Telomeric DNA in ALT cells is characterized by free telomeric circles and heterogeneous t-loops. Molecular and cellular biology. 2004;24(22):9948–57. Epub 2004/10/29. 10.1128/MCB.24.22.9948-9957.2004 ; PubMed Central PMCID: PMCPMC525488. 15509797 PMC525488

[11] Bryan TM, Englezou A, Gupta J, Bacchetti S, Reddel RR. Telomere elongation in immortal human cells without detectable telomerase activity. EMBO J. 1995;14(17):4240–8. Epub 1995/09/01. ; PubMed Central PMCID: PMCPMC394507. 7556065 10.1002/j.1460-2075.1995.tb00098.x PMC394507

[12] Heaphy CM, de Wilde RF, Jiao Y, Klein AP, Edil BH, Shi C, et al Altered telomeres in tumors with ATRX and DAXX mutations. Science. 2011;333(6041):425 10.1126/science.1207313 ; PubMed Central PMCID: PMCPMC3174141. 21719641 PMC3174141

[13] Jiao Y, Killela PJ, Reitman ZJ, Rasheed AB, Heaphy CM, de Wilde RF, et al Frequent ATRX, CIC, FUBP1 and IDH1 mutations refine the classification of malignant gliomas. Oncotarget. 2012;3(7):709–22. doi: 10.18632/oncotarget.588 ; PubMed Central PMCID: PMCPMC3443254. 22869205 10.18632/oncotarget.588 PMC3443254

[14] Jiao Y, Shi C, Edil BH, de Wilde RF, Klimstra DS, Maitra A, et al DAXX/ATRX, MEN1, and mTOR pathway genes are frequently altered in pancreatic neuroendocrine tumors. Science. 2011;331(6021):1199–203. 10.1126/science.1200609 ; PubMed Central PMCID: PMCPMC3144496. 21252315 PMC3144496

[15] Killela PJ, Reitman ZJ, Jiao Y, Bettegowda C, Agrawal N, Diaz LA Jr., et al TERT promoter mutations occur frequently in gliomas and a subset of tumors derived from cells with low rates of self-renewal. Proc Natl Acad Sci U S A. 2013;110(15):6021–6. 10.1073/pnas.1303607110 ; PubMed Central PMCID: PMCPMC3625331. 23530248 PMC3625331

[16] Schwartzentruber J, Korshunov A, Liu XY, Jones DT, Pfaff E, Jacob K, et al Driver mutations in histone H3.3 and chromatin remodelling genes in paediatric glioblastoma. Nature. 2012;482(7384):226–31. 10.1038/nature10833 . 22286061

[17] Xue Y, Gibbons R, Yan Z, Yang D, McDowell TL, Sechi S, et al The ATRX syndrome protein forms a chromatin-remodeling complex with Daxx and localizes in promyelocytic leukemia nuclear bodies. Proc Natl Acad Sci U S A. 2003;100(19):10635–40. Epub 2003/09/04. 10.1073/pnas.1937626100 ; PubMed Central PMCID: PMCPMC196856. 12953102 PMC196856

[18] Drane P, Ouararhni K, Depaux A, Shuaib M, Hamiche A. The death-associated protein DAXX is a novel histone chaperone involved in the replication-independent deposition of H3.3. Genes Dev. 2010;24(12):1253–65. Epub 2010/05/28. 10.1101/gad.566910 ; PubMed Central PMCID: PMCPMC2885661. 20504901 PMC2885661

[19] Lewis PW, Elsaesser SJ, Noh KM, Stadler SC, Allis CD. Daxx is an H3.3-specific histone chaperone and cooperates with ATRX in replication-independent chromatin assembly at telomeres. Proc Natl Acad Sci U S A. 2010;107(32):14075–80. 10.1073/pnas.1008850107 ; PubMed Central PMCID: PMCPMC2922592. 20651253 PMC2922592

[20] Goldberg AD, Banaszynski LA, Noh KM, Lewis PW, Elsaesser SJ, Stadler S, et al Distinct factors control histone variant H3.3 localization at specific genomic regions. Cell. 2010;140(5):678–91. 10.1016/j.cell.2010.01.003 ; PubMed Central PMCID: PMCPMC2885838. 20211137 PMC2885838

[21] Udugama M, FT MC, Chan FL, Tang MC, Pickett HA, JD RM, et al Histone variant H3.3 provides the heterochromatic H3 lysine 9 tri-methylation mark at telomeres. Nucleic Acids Res. 2015;43(21):10227–37. Epub 2015/08/26. 10.1093/nar/gkv847 ; PubMed Central PMCID: PMCPMC4666390. 26304540 PMC4666390

[22] Wong LH, McGhie JD, Sim M, Anderson MA, Ahn S, Hannan RD, et al ATRX interacts with H3.3 in maintaining telomere structural integrity in pluripotent embryonic stem cells. Genome Res. 2010;20(3):351–60. Epub 2010/01/30. 10.1101/gr.101477.109 ; PubMed Central PMCID: PMCPMC2840985. 20110566 PMC2840985

[23] O'Sullivan RJ, Arnoult N, Lackner DH, Oganesian L, Haggblom C, Corpet A, et al Rapid induction of alternative lengthening of telomeres by depletion of the histone chaperone ASF1. Nat Struct Mol Biol. 2014;21(2):167–74. Epub 2014/01/15. 10.1038/nsmb.2754 ; PubMed Central PMCID: PMCPMC3946341. 24413054 PMC3946341

[24] Lovejoy CA, Li W, Reisenweber S, Thongthip S, Bruno J, de Lange T, et al Loss of ATRX, genome instability, and an altered DNA damage response are hallmarks of the alternative lengthening of telomeres pathway. PLoS Genet. 2012;8(7):e1002772 10.1371/journal.pgen.1002772 ; PubMed Central PMCID: PMCPMC3400581. 22829774 PMC3400581

[25] Napier CE, Huschtscha LI, Harvey A, Bower K, Noble JR, Hendrickson EA, et al ATRX represses alternative lengthening of telomeres. Oncotarget. 2015;6(18):16543–58. Epub 2015/05/23. doi: 10.18632/oncotarget.3846 . 26001292 10.18632/oncotarget.3846 PMC4599288

[26] Clynes D, Jelinska C, Xella B, Ayyub H, Scott C, Mitson M, et al Suppression of the alternative lengthening of telomere pathway by the chromatin remodelling factor ATRX. Nature communications. 2015;6:7538 Epub 2015/07/07. 10.1038/ncomms8538 ; PubMed Central PMCID: PMCPmc4501375. 26143912 PMC4501375

[27] Clynes D, Jelinska C, Xella B, Ayyub H, Taylor S, Mitson M, et al ATRX dysfunction induces replication defects in primary mouse cells. PLoS One. 2014;9(3):e92915 Epub 2014/03/22. 10.1371/journal.pone.0092915 ; PubMed Central PMCID: PMCPMC3961441. 24651726 PMC3961441

[28] Eid R, Demattei MV, Episkopou H, Auge-Gouillou C, Decottignies A, Grandin N, et al Genetic Inactivation of ATRX Leads to a Decrease in the Amount of Telomeric Cohesin and Level of Telomere Transcription in Human Glioma Cells. Molecular and cellular biology. 2015;35(16):2818–30. Epub 2015/06/10. 10.1128/MCB.01317-14 ; PubMed Central PMCID: PMCPmc4508314. 26055325 PMC4508314

[29] Flynn RL, Cox KE, Jeitany M, Wakimoto H, Bryll AR, Ganem NJ, et al Alternative lengthening of telomeres renders cancer cells hypersensitive to ATR inhibitors. Science. 2015;347(6219):273–7. 10.1126/science.1257216 ; PubMed Central PMCID: PMCPMC4358324. 25593184 PMC4358324

[30] Nguyen DN, Heaphy CM, de Wilde RF, Orr BA, Odia Y, Eberhart CG, et al Molecular and morphologic correlates of the alternative lengthening of telomeres phenotype in high-grade astrocytomas. Brain Pathol. 2013;23(3):237–43. 10.1111/j.1750-3639.2012.00630.x ; PubMed Central PMCID: PMCPMC3827727. 22928601 PMC3827727

[31] Abedalthagafi M, Phillips JJ, Kim GE, Mueller S, Haas-Kogen DA, Marshall RE, et al The alternative lengthening of telomere phenotype is significantly associated with loss of ATRX expression in high-grade pediatric and adult astrocytomas: a multi-institutional study of 214 astrocytomas. Mod Pathol. 2013;26(11):1425–32. 10.1038/modpathol.2013.90 . 23765250 PMC6054791

[32] Hakin-Smith V, Jellinek DA, Levy D, Carroll T, Teo M, Timperley WR, et al Alternative lengthening of telomeres and survival in patients with glioblastoma multiforme. Lancet. 2003;361(9360):836–8. Epub 2003/03/19. . 12642053 10.1016/s0140-6736(03)12681-5

[33] McDonald KL, McDonnell J, Muntoni A, Henson JD, Hegi ME, von Deimling A, et al Presence of alternative lengthening of telomeres mechanism in patients with glioblastoma identifies a less aggressive tumor type with longer survival. J Neuropathol Exp Neurol. 2010;69(7):729–36. Epub 2010/06/11. 10.1097/NEN.0b013e3181e576cf . 20535033

[34] Mangerel J, Price A, Castelo-Branco P, Brzezinski J, Buczkowicz P, Rakopoulos P, et al Alternative lengthening of telomeres is enriched in, and impacts survival of TP53 mutant pediatric malignant brain tumors. Acta Neuropathol. 2014;128(6):853–62. 10.1007/s00401-014-1348-1 . 25315281

[35] Barretina J, Caponigro G, Stransky N, Venkatesan K, Margolin AA, Kim S, et al The Cancer Cell Line Encyclopedia enables predictive modelling of anticancer drug sensitivity. Nature. 2012;483(7391):603–7. Epub 2012/03/31. 10.1038/nature11003 ; PubMed Central PMCID: PMCPMC3320027. 22460905 PMC3320027

[36] Gao J, Aksoy BA, Dogrusoz U, Dresdner G, Gross B, Sumer SO, et al Integrative analysis of complex cancer genomics and clinical profiles using the cBioPortal. Sci Signal. 2013;6(269):pl1 Epub 2013/04/04. 10.1126/scisignal.2004088 ; PubMed Central PMCID: PMCPMC4160307. 23550210 PMC4160307

[37] Cerami E, Gao J, Dogrusoz U, Gross BE, Sumer SO, Aksoy BA, et al The cBio cancer genomics portal: an open platform for exploring multidimensional cancer genomics data. Cancer Discov. 2012;2(5):401–4. Epub 2012/05/17. 10.1158/2159-8290.CD-12-0095 ; PubMed Central PMCID: PMCPMC3956037. 22588877 PMC3956037

[38] O'Connor PM, Jackman J, Bae I, Myers TG, Fan S, Mutoh M, et al Characterization of the p53 tumor suppressor pathway in cell lines of the National Cancer Institute anticancer drug screen and correlations with the growth-inhibitory potency of 123 anticancer agents. Cancer Res. 1997;57(19):4285–300. Epub 1997/10/23 22:22. . 9331090

[39] Xu J, Erdreich-Epstein A, Gonzalez-Gomez I, Melendez EY, Smbatyan G, Moats RA, et al Novel cell lines established from pediatric brain tumors. J Neurooncol. 2012;107(2):269–80. 10.1007/s11060-011-0756-5 ; PubMed Central PMCID: PMCPMC3379550. 22120608 PMC3379550

[40] Bax DA, Little SE, Gaspar N, Perryman L, Marshall L, Viana-Pereira M, et al Molecular and phenotypic characterisation of paediatric glioma cell lines as models for preclinical drug development. PLoS One. 2009;4(4):e5209 Epub 2009/04/15. 10.1371/journal.pone.0005209 ; PubMed Central PMCID: PMCPMC2666263. 19365568 PMC2666263

[41] Ishii N, Maier D, Merlo A, Tada M, Sawamura Y, Diserens AC, et al Frequent co-alterations of TP53, p16/CDKN2A, p14ARF, PTEN tumor suppressor genes in human glioma cell lines. Brain Pathol. 1999;9(3):469–79. Epub 1999/07/23. . 10416987 10.1111/j.1750-3639.1999.tb00536.x PMC8098486

[42] Levesley J, Steele L, Taylor C, Sinha P, Lawler SE. ABT-263 enhances sensitivity to metformin and 2-deoxyglucose in pediatric glioma by promoting apoptotic cell death. PLoS One. 2013;8(5):e64051 Epub 2013/05/22. 10.1371/journal.pone.0064051 ; PubMed Central PMCID: PMCPMC3656874. 23691145 PMC3656874

[43] Scheel C, Schaefer KL, Jauch A, Keller M, Wai D, Brinkschmidt C, et al Alternative lengthening of telomeres is associated with chromosomal instability in osteosarcomas. Oncogene. 2001;20(29):3835–44. Epub 2001/07/06. 10.1038/sj.onc.1204493 . 11439347

[44] Bjerke L, Mackay A, Nandhabalan M, Burford A, Jury A, Popov S, et al Histone H3.3. mutations drive pediatric glioblastoma through upregulation of MYCN. Cancer Discov. 2013;3(5):512–9. Epub 2013/03/30. 10.1158/2159-8290.CD-12-0426 ; PubMed Central PMCID: PMCPMC3763966. 23539269 PMC3763966

[45] Lau LM, Dagg RA, Henson JD, Au AY, Royds JA, Reddel RR. Detection of alternative lengthening of telomeres by telomere quantitative PCR. Nucleic Acids Res. 2013;41(2):e34 Epub 2012/08/28. 10.1093/nar/gks781 ; PubMed Central PMCID: PMCPMC3553966. 22923525 PMC3553966

[46] Pampliega O, Orhon I, Patel B, Sridhar S, Diaz-Carretero A, Beau I, et al Functional interaction between autophagy and ciliogenesis. Nature. 2013;502(7470):194–200. Epub 2013/10/04. 10.1038/nature12639 ; PubMed Central PMCID: PMCPMC3896125. 24089209 PMC3896125

[47] Mukherjee J, Johannessen TC, Ohba S, Chow TT, Jones L, Pandita A, et al Mutant IDH1 Cooperates with ATRX Loss to Drive the Alternative Lengthening of Telomere Phenotype in Glioma. Cancer Res. 2018;78(11):2966–77. Epub 2018/03/17. 10.1158/0008-5472.CAN-17-2269 . 29545335 PMC10578296

[48] Min J, Wright WE, Shay JW. Alternative lengthening of telomeres can be maintained by preferential elongation of lagging strands. Nucleic Acids Res. 2017;45(5):2615–28. Epub 2017/01/14. 10.1093/nar/gkw1295 ; PubMed Central PMCID: PMCPMC5389697. 28082393 PMC5389697

[49] Perrem K, Colgin LM, Neumann AA, Yeager TR, Reddel RR. Coexistence of alternative lengthening of telomeres and telomerase in hTERT-transfected GM847 cells. Molecular and cellular biology. 2001;21(12):3862–75. Epub 2001/05/22. 10.1128/MCB.21.12.3862-3875.2001 ; PubMed Central PMCID: PMCPMC87050. 11359895 PMC87050

[50] Chang FT, McGhie JD, Chan FL, Tang MC, Anderson MA, Mann JR, et al PML bodies provide an important platform for the maintenance of telomeric chromatin integrity in embryonic stem cells. Nucleic Acids Res. 2013;41(8):4447–58. Epub 2013/02/28. 10.1093/nar/gkt114 ; PubMed Central PMCID: PMCPMC3632112. 23444137 PMC3632112

[51] Neumann AA, Watson CM, Noble JR, Pickett HA, Tam PP, Reddel RR. Alternative lengthening of telomeres in normal mammalian somatic cells. Genes Dev. 2013;27(1):18–23. Epub 2013/01/12. 10.1101/gad.205062.112 ; PubMed Central PMCID: PMCPMC3553280. 23307865 PMC3553280

[52] Pickett HA, Reddel RR. The role of telomere trimming in normal telomere length dynamics. Cell Cycle. 2012;11(7):1309–15. Epub 2012/03/17. 10.4161/cc.19632 . 22421147

[53] Pickett HA, Cesare AJ, Johnston RL, Neumann AA, Reddel RR. Control of telomere length by a trimming mechanism that involves generation of t-circles. EMBO J. 2009;28(7):799–809. Epub 2009/02/14. 10.1038/emboj.2009.42 ; PubMed Central PMCID: PMCPMC2670870. 19214183 PMC2670870

[54] Rivera T, Haggblom C, Cosconati S, Karlseder J. A balance between elongation and trimming regulates telomere stability in stem cells. Nat Struct Mol Biol. 2017;24(1):30–9. Epub 2016/12/06. 10.1038/nsmb.3335 ; PubMed Central PMCID: PMCPMC5215970. 27918544 PMC5215970

[55] Fakhoury J, Marie-Egyptienne DT, Londono-Vallejo JA, Autexier C. Telomeric function of mammalian telomerases at short telomeres. J Cell Sci. 2010;123(Pt 10):1693–704. Epub 2010/04/30. 10.1242/jcs.063636 . 20427319

[56] Louis DN, Perry A, Reifenberger G, von Deimling A, Figarella-Branger D, Cavenee WK, et al The 2016 World Health Organization Classification of Tumors of the Central Nervous System: a summary. Acta Neuropathol. 2016;131(6):803–20. Epub 2016/05/10. 10.1007/s00401-016-1545-1 . 27157931

[57] Wiestler B, Capper D, Holland-Letz T, Korshunov A, von Deimling A, Pfister SM, et al ATRX loss refines the classification of anaplastic gliomas and identifies a subgroup of IDH mutant astrocytic tumors with better prognosis. Acta Neuropathol. 2013;126(3):443–51. Epub 2013/08/02. 10.1007/s00401-013-1156-z . 23904111

[58] Koschmann C, Calinescu AA, Nunez FJ, Mackay A, Fazal-Salom J, Thomas D, et al ATRX loss promotes tumor growth and impairs nonhomologous end joining DNA repair in glioma. Sci Transl Med. 2016;8(328):328ra28 Epub 2016/03/05. 10.1126/scitranslmed.aac8228 ; PubMed Central PMCID: PMCPMC5381643. 26936505 PMC5381643

[59] Mender I, Shay JW. Telomerase Repeated Amplification Protocol (TRAP). Bio Protoc. 2015;5(22). Epub 2016/05/18. ; PubMed Central PMCID: PMCPMC4863463. 27182535 10.21769/bioprotoc.1657 PMC4863463

[60] Schneider CA, Rasband WS, Eliceiri KW. NIH Image to ImageJ: 25 years of image analysis. Nat Methods. 2012;9(7):671–5. Epub 2012/08/30. ; PubMed Central PMCID: PMCPMC5554542. 22930834 10.1038/nmeth.2089 PMC5554542

[61] Schindelin J, Arganda-Carreras I, Frise E, Kaynig V, Longair M, Pietzsch T, et al Fiji: an open-source platform for biological-image analysis. Nat Methods. 2012;9(7):676–82. Epub 2012/06/30. 10.1038/nmeth.2019 ; PubMed Central PMCID: PMCPMC3855844. 22743772 PMC3855844

[62] Henson JD, Cao Y, Huschtscha LI, Chang AC, Au AY, Pickett HA, et al DNA C-circles are specific and quantifiable markers of alternative-lengthening-of-telomeres activity. Nat Biotechnol. 2009;27(12):1181–5. 10.1038/nbt.1587 . 19935656

[63] Meeker AK, Gage WR, Hicks JL, Simon I, Coffman JR, Platz EA, et al Telomere length assessment in human archival tissues: combined telomere fluorescence in situ hybridization and immunostaining. Am J Pathol. 2002;160(4):1259–68. Epub 2002/04/12. 10.1016/S0002-9440(10)62553-9 ; PubMed Central PMCID: PMCPMC1867217. 11943711 PMC1867217

[64] Hsu PD, Scott DA, Weinstein JA, Ran FA, Konermann S, Agarwala V, et al DNA targeting specificity of RNA-guided Cas9 nucleases. Nat Biotechnol. 2013;31(9):827–32. Epub 2013/07/23. 10.1038/nbt.2647 ; PubMed Central PMCID: PMCPMC3969858. 23873081 PMC3969858

[65] Ran FA, Hsu PD, Lin CY, Gootenberg JS, Konermann S, Trevino AE, et al Double nicking by RNA-guided CRISPR Cas9 for enhanced genome editing specificity. Cell. 2013;154(6):1380–9. Epub 2013/09/03. 10.1016/j.cell.2013.08.021 ; PubMed Central PMCID: PMCPMC3856256. 23992846 PMC3856256

[66] Baker SJ, Markowitz S, Fearon ER, Willson JK, Vogelstein B. Suppression of human colorectal carcinoma cell growth by wild-type p53. Science. 1990;249(4971):912–5. Epub 1990/08/24. . 2144057 10.1126/science.2144057

[67] R Core Team. R: A language and environment for statistical computing. Vienna, Austria: R Foundation for Statistical Computing; 2015.

