# Supplementary material for: ATRX loss induces multiple hallmarks of the alternative lengthening of telomeres (ALT) phenotype in human glioma cell lines in a cell line-specific manner
Source: PLoS One. 2018 Sep 18;13(9):e0204159. doi: 10.1371/journal.pone.0204159 (PMC6143253; doi:10.1371/journal.pone.0204159)
Supplement: S1 Table — PCR primer sequences and associated PCR amplification conditions. (DOCX) [file pone.0204159.s001.docx]

| **S1 Table** | | |
| --- | --- | --- |
|  | **Primer sequence (5’-3’)** | **Cycling conditions** |
| *TERT* promoter [1] | Forward:  TGTAAAACGACGGCCAGTGGCCGATTCGACCTCTCT  Reverse:  AGCACCTCGCGGTAGTGG | 1. 96^o^C 2 min 2. 96^o^C 10 sec 3. 64^o^C 10 sec 4. 70^o^C 30 sec 5. Goto (2) 2X 6. 96^o^C 10 sec 7. 61^o^C 10 sec 8. 70^o^C 30 sec 9. Goto (2) 2X 10. 96^o^C 10 sec 11. 58^o^C 10 sec 12. 70^o^C 30 sec 13. Goto (2) 2X 14. 96^o^C 10 sec 15. 57^o^C 10 sec 16. 70^o^C 30 sec 17. Goto (2) 40X 18. 70^o^C 5 min |
| *IDH1* | Forward:  TGTAAAACGACGGCCAGTTGCCATCACTGCAGTTGTAG  Reverse:  TCACATTATTGCCAACATGACTT | 1. 95^o^C 5 min 2. 95^o^C 30 sec 3. 54.1^o^C 30 sec 4. 72^o^C 30 sec 5. Goto (2) 39X 6. 72^o^C 10 min |
| *IDH2* | Forward:  TGTAAAACGACGGCCAGTGGGTTCAAATTCTGGTTGAA  Reverse:  CAGGAAACAGCTATGACCTAGGCGAGGAGCTCCAGT | 1. 95^o^C 5 min 2. 95^o^C 30 sec 3. 60^o^C 30 sec 4. 72^o^C 30 sec 5. Goto (2) 39X 6. 72^o^C 10 min |
| *H3F3A* | Forward:  TGTAAAACGACGGCCAGTGGTGATCGTGGCAGGAAAAG  Reverse:  CCTCCAGGTAAGATTATGGCTTC | 1. 95^o^C 5 min 2. 95^o^C 30 sec 3. 54.1^o^C 30 sec 4. 72^o^C 30 sec 5. Goto (2) 39X 6. 72^o^C 10 min |
| *ATRX* exons 8-9 | Forward:  TGTAAAACGACGGCCAGTAGCCGTGACTCAGATGGAAT  Reverse:  CAGGAAACAGCTATGACCCAACAAAGGCTCTGGGTGAC | 1. 95^o^C 5 min 2. 95^o^C 30 sec 3. 58.4^o^C 30 sec 4. 72^o^C 30 sec 5. Goto (2) 39X 6. 72^o^C 10 min |
| *TP53* (R273H sequencing) | Forward:  TGTAAAACGACGGCCAGTGGCTCTGACTGTACCACCAT  Reverse:  CAGGAAACAGCTATGACCCTCCCCTTTCTTGCGGAGA | 1. 95^o^C 5 min 2. 95^o^C 30 sec 3. 58.4^o^C 95^o^C 4. 72^o^C 30 sec 5. Goto (2) 39X 6. 72^o^C 10 min |
| *TP53* (exons 5-6) [2] | Forward:  TGTAAAACGACGGCCAGTGTTTCTTTGCTGCCGTCTTC  Reverse:  CAGGAAACAGCTATGACCCCCTACTGCTCACCTGGAG | 1. 94^o^C 2 min 2. 94^o^C 40 sec 3. 55.5^o^C 40 sec 4. 72^o^C 1 min 5. Goto (2) 39X 6. 72^o^C 2 min |
| *TP53* (exon 7) [2] | Forward:  TGTAAAACGACGGCCAGTTGCTTGCCACAGGTCTCC  Reverse:  CAGGAAACAGCTATGACCGGTCAGAGGCAAGCAGAGG | 1. 94^o^C 2 min 2. 94^o^C 40 sec 3. 58.5^o^C 40 sec 4. 72^o^C 1 min 5. Goto (2) 40X 6. 72^o^C 1 min |
| *TP53* (exons 8-9) [2] | Forward:  TGTAAAACGACGGCCAGTGGGAGTAGATGGAGCCTGGT  Reverse:  CAGGAAACAGCTATGACCTGTCTTTGAGGCATCACTGC | 1. 94^o^C 2 min 2. 94^o^C 40 sec 3. 58^o^C 40 sec 4. 72^o^C 1 min 5. Goto (2) 10X 6. 94^o^C 40 sec 7. 60^o^C 40 sec 8. 72^o^C 1 min 9. Goto (2) 10X 10. 94^o^C 40 sec 11. 62^o^C 40 sec 12. 72^o^C 1 min 13. Goto (2) 10X 14. 94^o^C 40 sec 15. 64^o^C 40 sec 16. 72^o^C 1 min 17. Goto (2) 10X 18. 72^o^C 2 min |
| *TP53* (exon 10) | Forward:  TGTAAAACGACGGCCAGTGCAACAGAGTGAGACCCCAT  Reverse:  CAGGAAACAGCTATGACCTGAAGGCAGGATGAGAATGGA | 1. 95^o^C 5 min 2. 95^o^C 30 sec 3. 58.4^o^C 95^o^C 4. 72^o^C 30 sec 5. Goto (2) 39X 6. 72^o^C 10 min |

**References**:

1. Killela PJ, Reitman ZJ, Jiao Y, Bettegowda C, Agrawal N, Diaz LA, Jr., et al. TERT promoter mutations occur frequently in gliomas and a subset of tumors derived from cells with low rates of self-renewal. Proc Natl Acad Sci U S A. 2013;110(15):6021-6. doi: 10.1073/pnas.1303607110. PubMed PMID: 23530248; PubMed Central PMCID: PMCPMC3625331.

2. Yachida S, White CM, Naito Y, Zhong Y, Brosnan JA, Macgregor-Das AM, et al. Clinical significance of the genetic landscape of pancreatic cancer and implications for identification of potential long-term survivors. Clin Cancer Res. 2012;18(22):6339-47. Epub 2012/09/20. doi: 10.1158/1078-0432.CCR-12-1215. PubMed PMID: 22991414; PubMed Central PMCID: PMCPMC3500447.
