# Supplementary figures and images for: ATRX loss induces multiple hallmarks of the alternative lengthening of telomeres (ALT) phenotype in human glioma cell lines in a cell line-specific manner
Source: PLoS One. 2018 Sep 18;13(9):e0204159. doi: 10.1371/journal.pone.0204159 (PMC6143253; doi:10.1371/journal.pone.0204159)

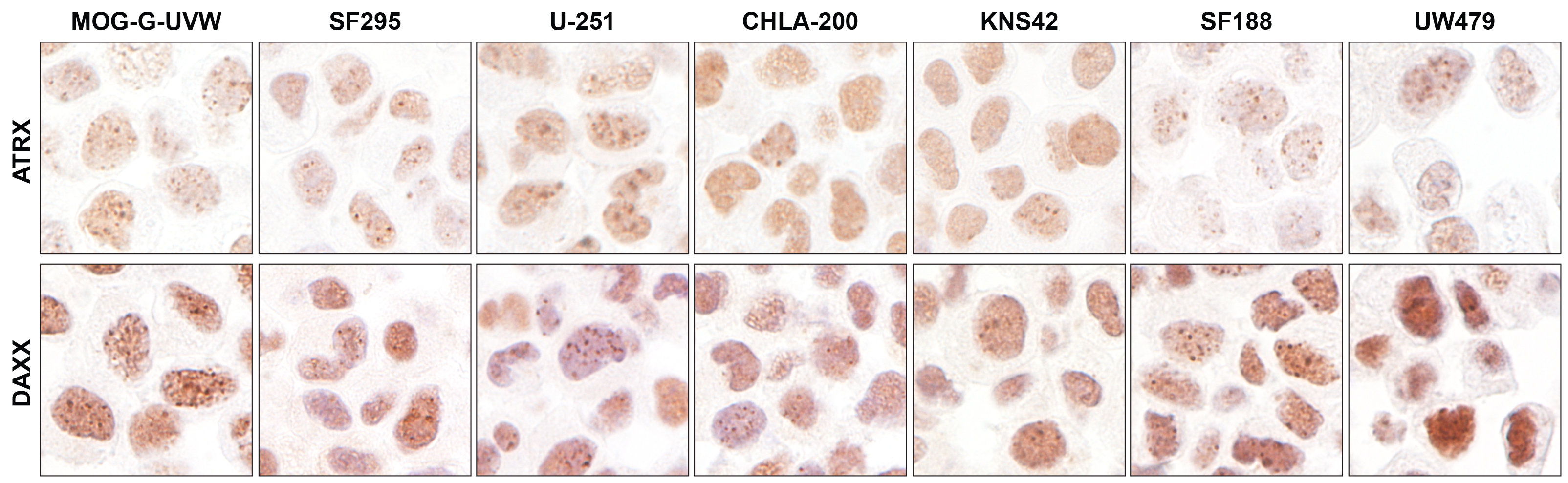

Supplement: S1 Fig — All glioma cell lines utilized in this study retain nuclear expression of both proteins, with formation of characteristic nuclear puncta. (TIF) [file pone.0204159.s002.tif]

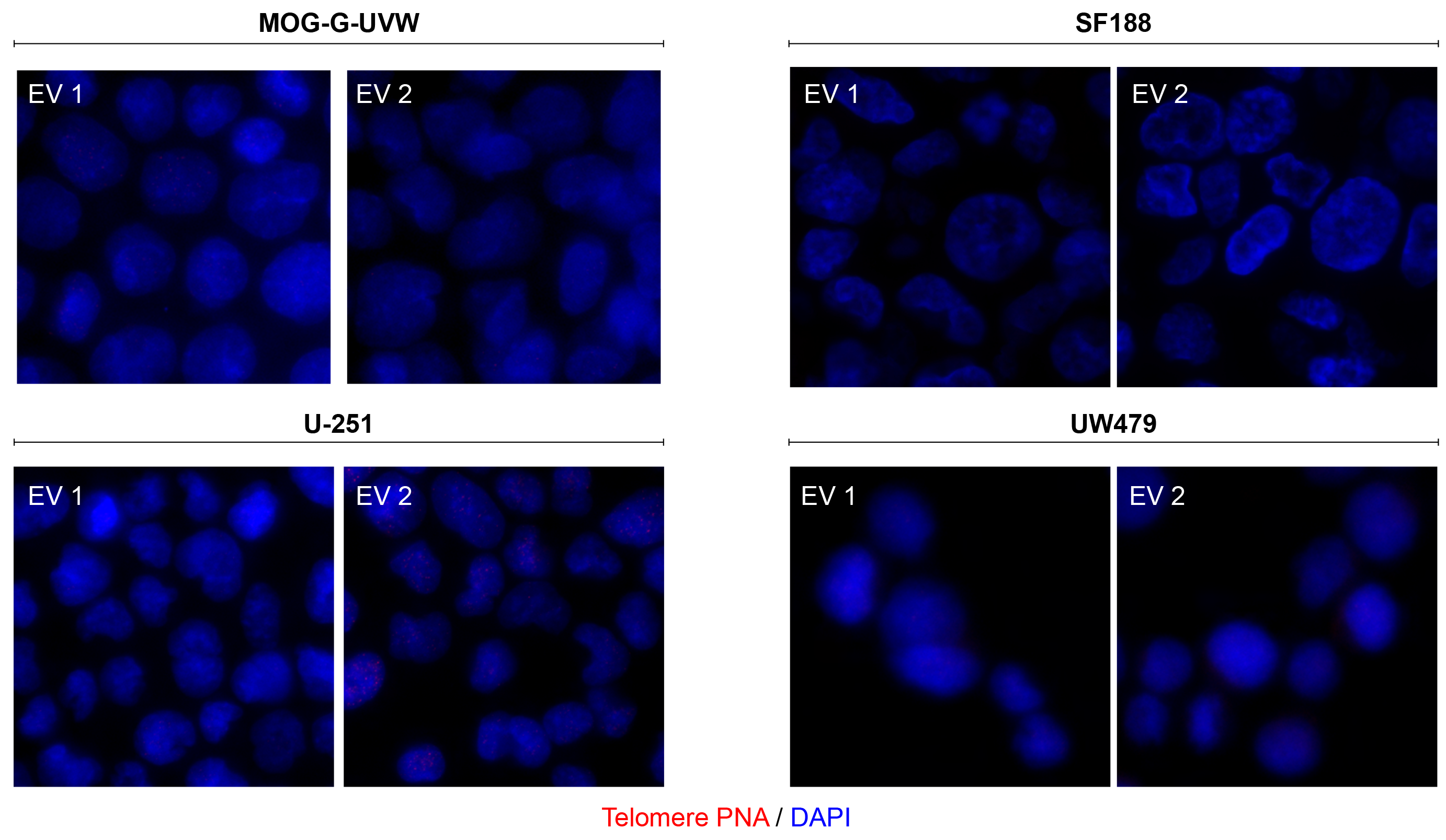

Supplement: S2 Fig — Representative images of telomere FISH from EV clones isolated from MOG-G-UVW, SF188, U-251 and UW479 indicate a lack of ALT-associated ultrabright telomere DNA foci. (TIF) [file pone.0204159.s003.tif]

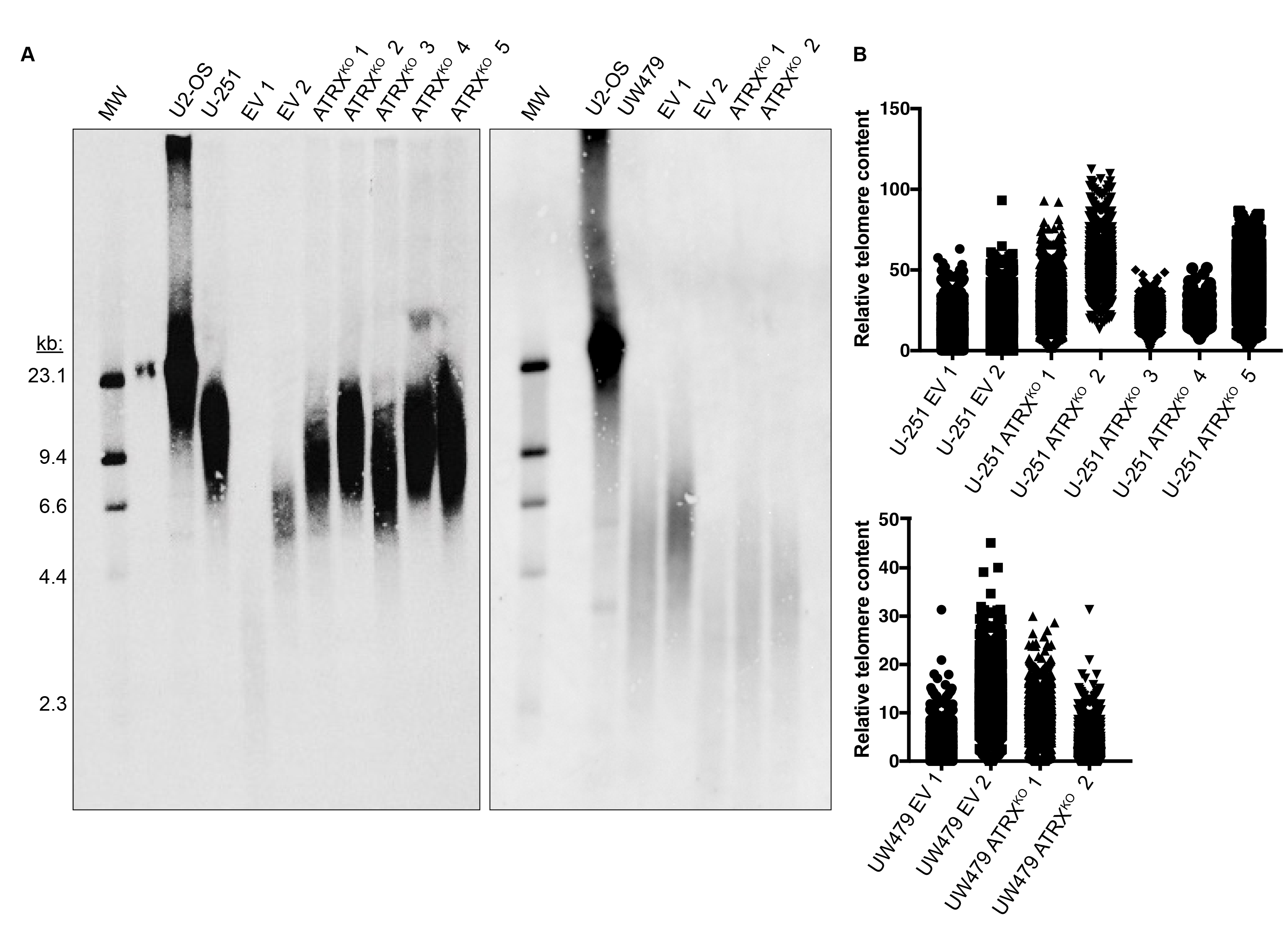

Supplement: S3 Fig — (A) TRF Southern blot analysis does not reveal gross changes in telomere lengths between parental, empty vector, and ATRXKO cells. (B) Measurement of telomere pixel intensity after quantitative telomere FISH does not reveal increased heterogeneity in overall telomere content between empty vector and ATRXKO cells. (TIF) [file pone.0204159.s004.tif]

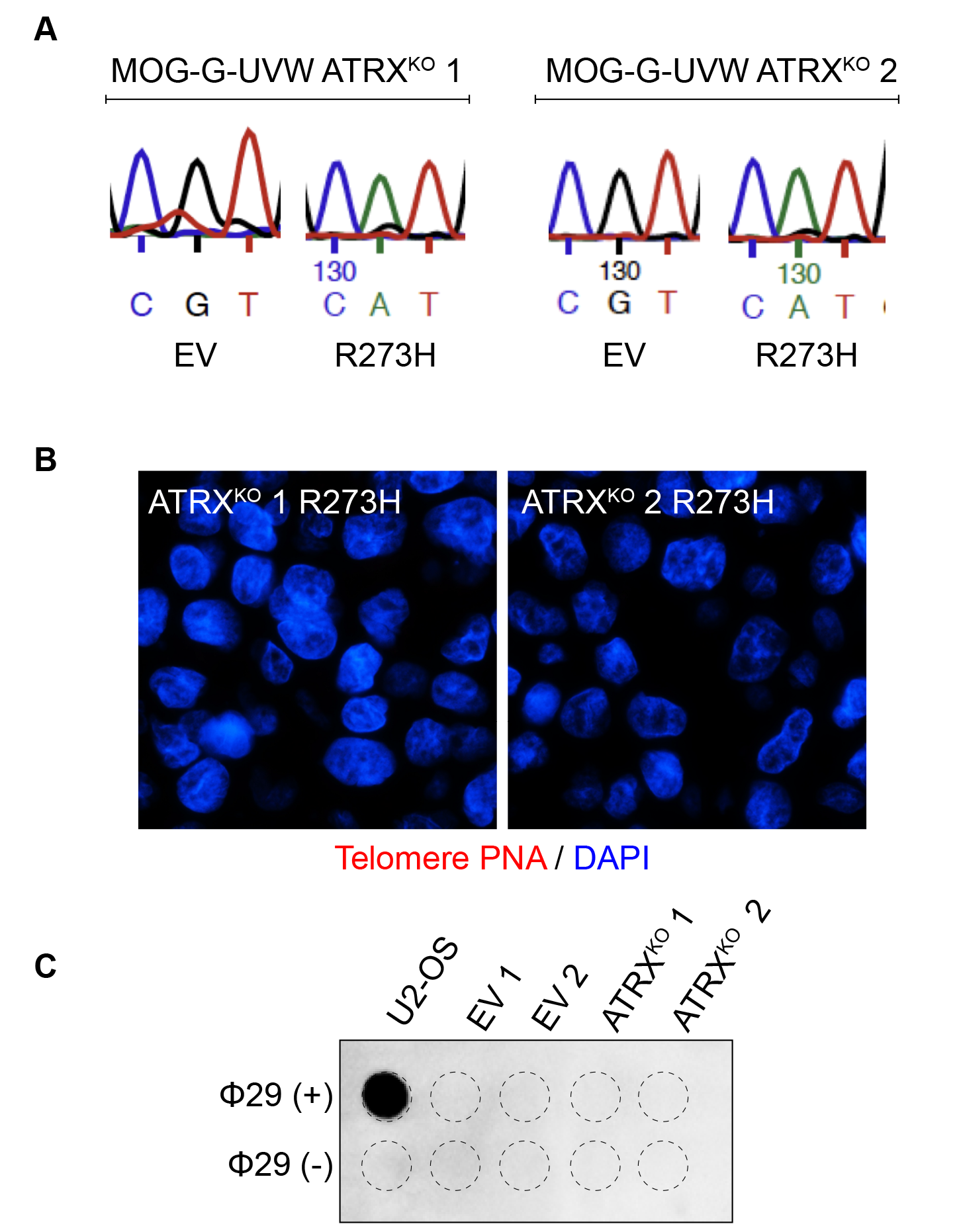

Supplement: S4 Fig — (A) The R273H dominant-negative variant of p53 was stably overexpressed in ATRX-knockout MOG-G-UVW cells. This mutation did not result in (B) ultrabright telomeric DNA foci or (C) c-circles. A smaller input of U2-OS DNA (30 ng, compared to 150 ng) included as a positive control. (TIF) [file pone.0204159.s005.tif]

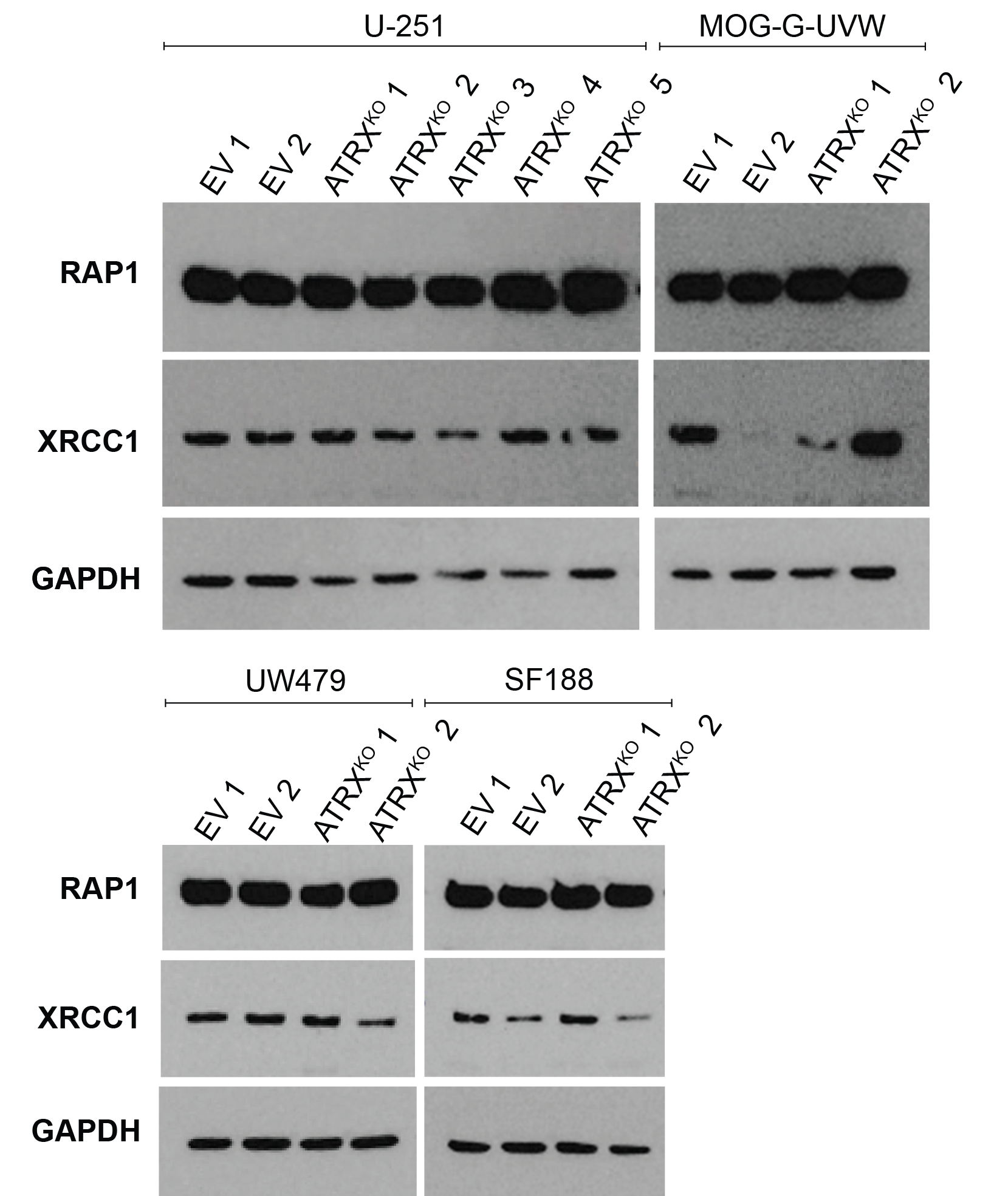

Supplement: S5 Fig — RAP1 and XRCC1 levels were assessed in EV and ATRXKO clones by immunoblotting. No consistent changes in expression of these proteins were observed after ATRX loss in clones showing ALT hallmarks. (TIF) [file pone.0204159.s006.tif]

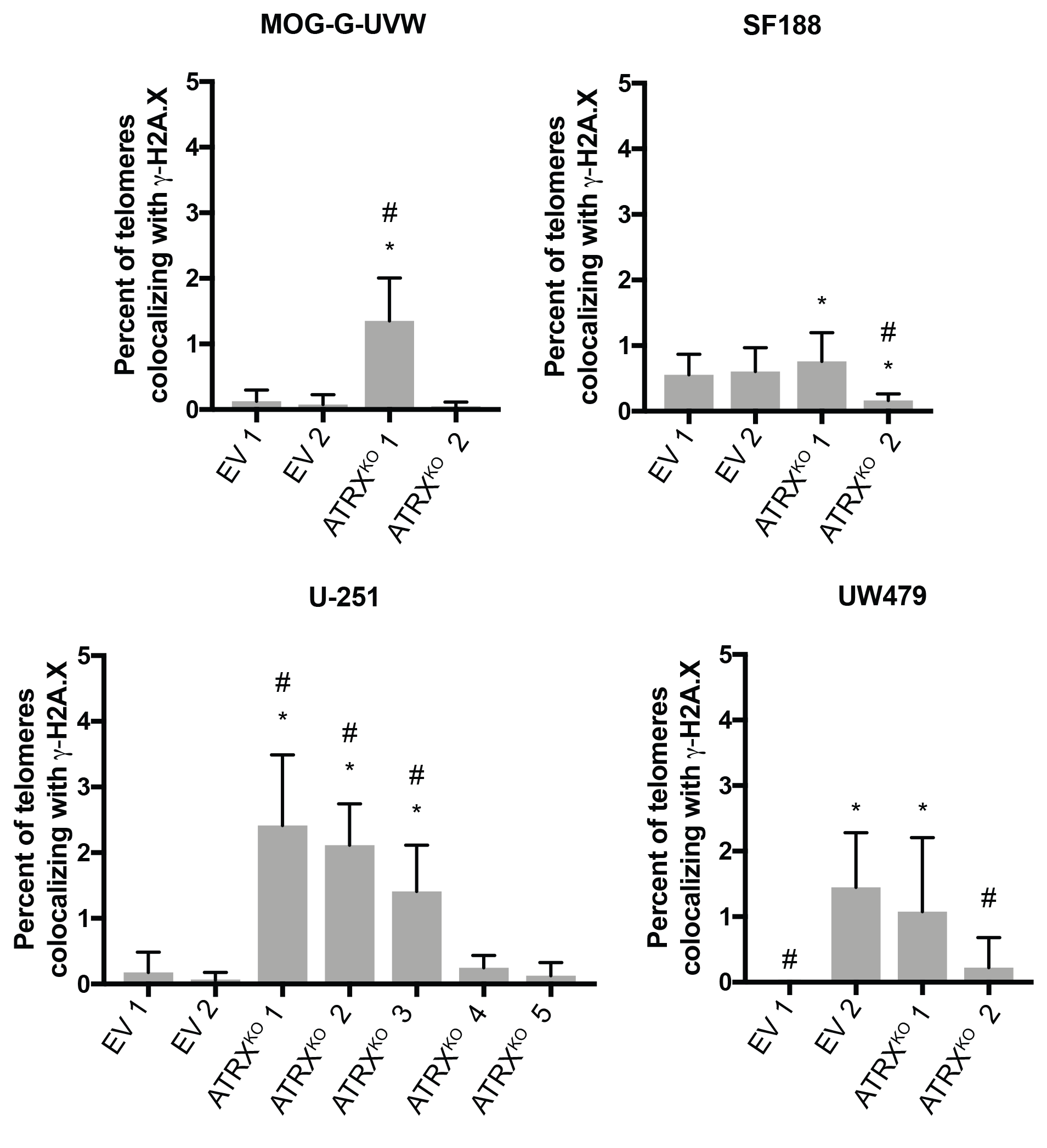

Supplement: S6 Fig — Combined telomere-specific FISH and immunofluorescence against phospho-H2A.X was performed in EV and ATRXKO clones, and 36 images (magnification = 400X) per experiment were obtained via scanning microscopy. A minimum of 2000 cells were analyzed for each clone. Telomeres and phospho-H2A.X puncta were identified by setting pixel intensity thresholds after background subtraction. Ultrabright telomeric foci and cells overexpressing phospho-H2A.X were excluded from analysis by eliminating signals larger than 20 pixels. Colocalization events were identified using the Image J Colocalization plugin [46], and percent colocalization was calculated as a fraction of total telomeres. Significance was calculated using a one-way ANOVA incorporating a Tukey’s multiple comparisons test. Asterisks (*) indicate significant difference from the EV1 clone, while pound signs (#) indicate significant difference from the EV2 clone. Error bars represent standard deviation. (TIF) [file pone.0204159.s007.tif]

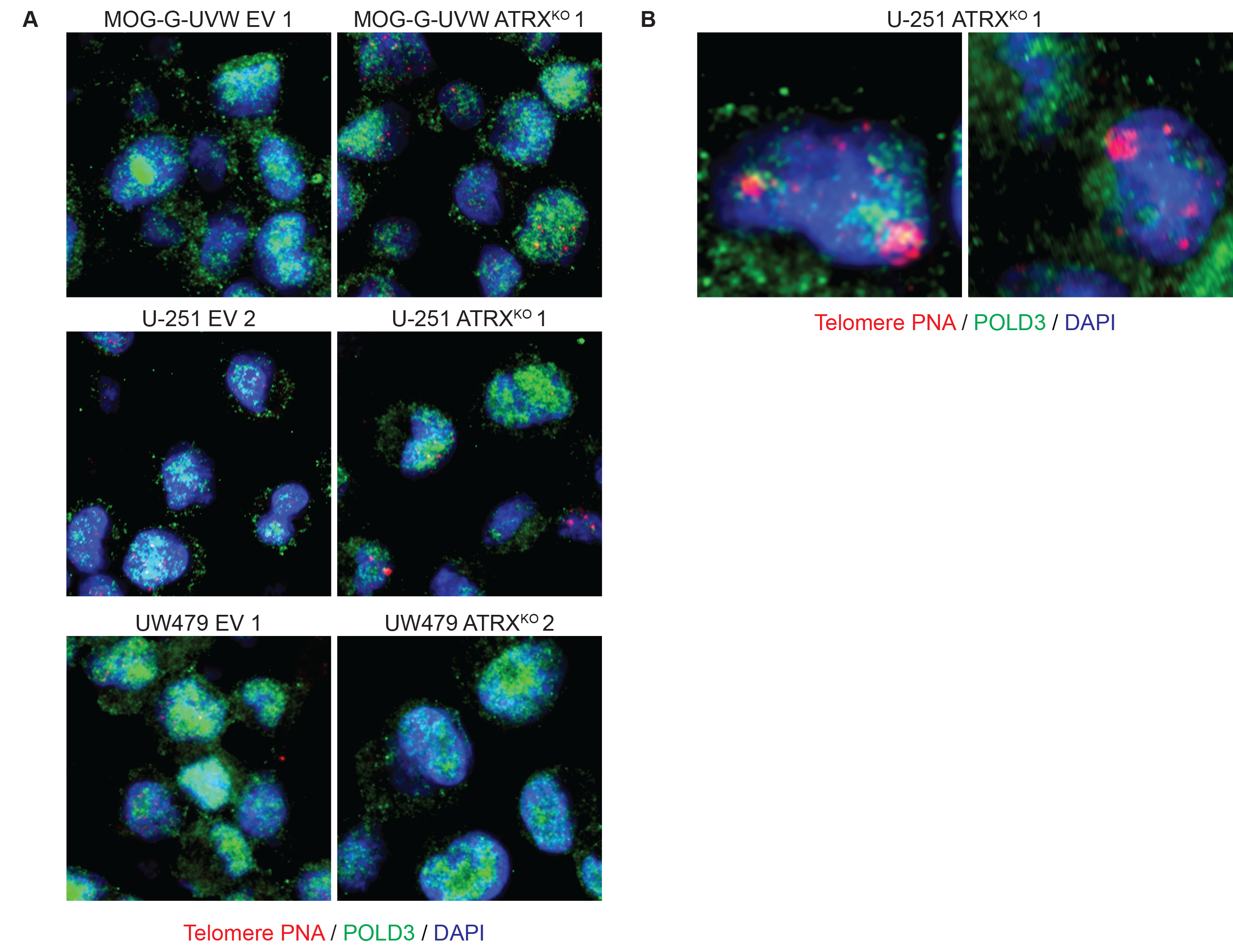

Supplement: S7 Fig — Combined telomere-specific FISH and immunofluorescence against POLD3 was performed in EV and ATRXKO. A) In both EV and ATRXKO clones, a pan-nuclear, speckled pattern was observed for POLD3. Representative images (magnification = 400X) for EV and ATRXKO clones from MOG-G-UVW, U-251, and UW479 are shown. B) No consistent pattern of colocalization between POLD3 and ALT-associated telomeric DNA foci was observed. Representative images (magnification = 400X) of cells from U-251 ATRXKO 1 are shown. (TIF) [file pone.0204159.s008.tif]

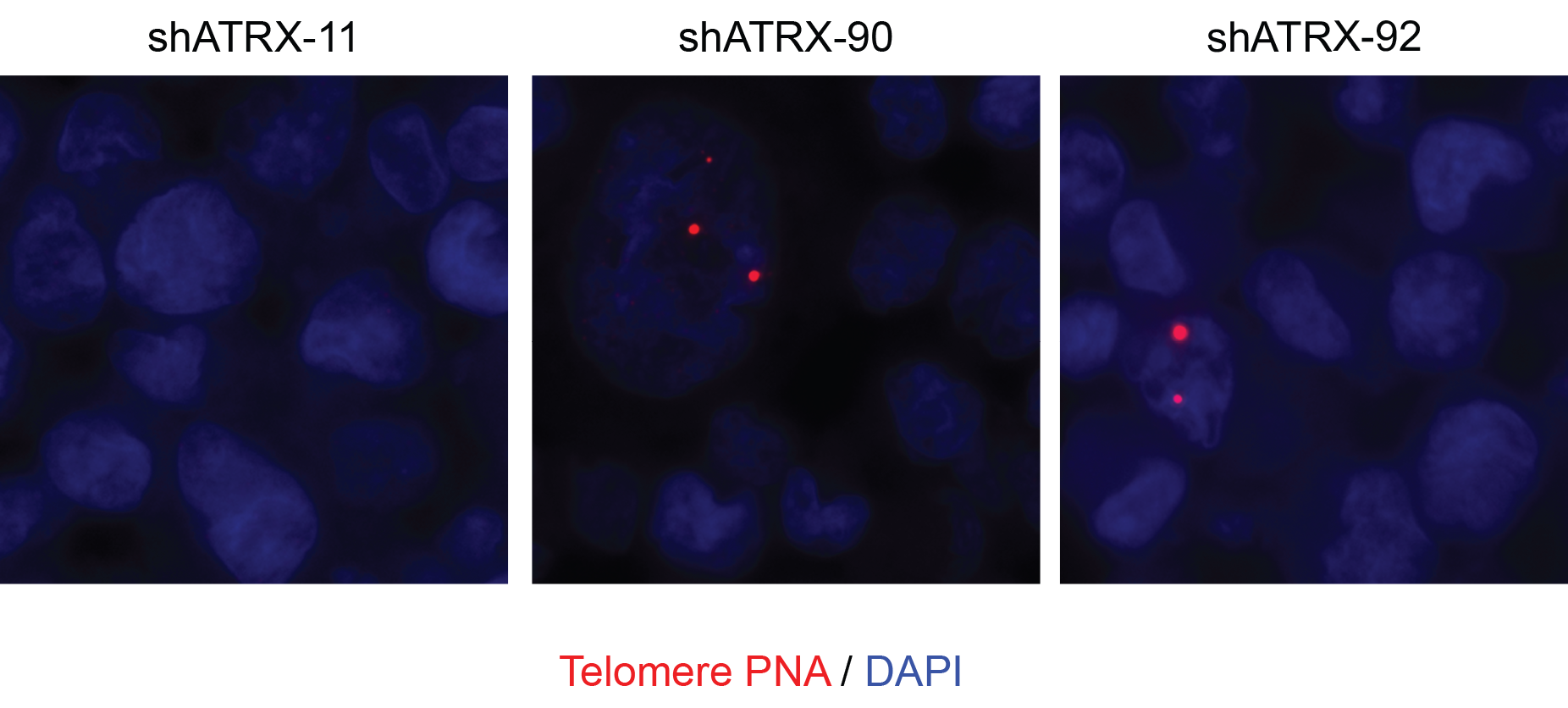

Supplement: S8 Fig — Representative telomere FISH from U-251 shATRX cells indicates that, while ultrabright telomeric DNA foci persist in U-251 shATRX-90 and U-251 shATRX-92, this ALT hallmark is no longer present in U-251 shATRX-11 after over ten passages. (TIF) [file pone.0204159.s009.tif]

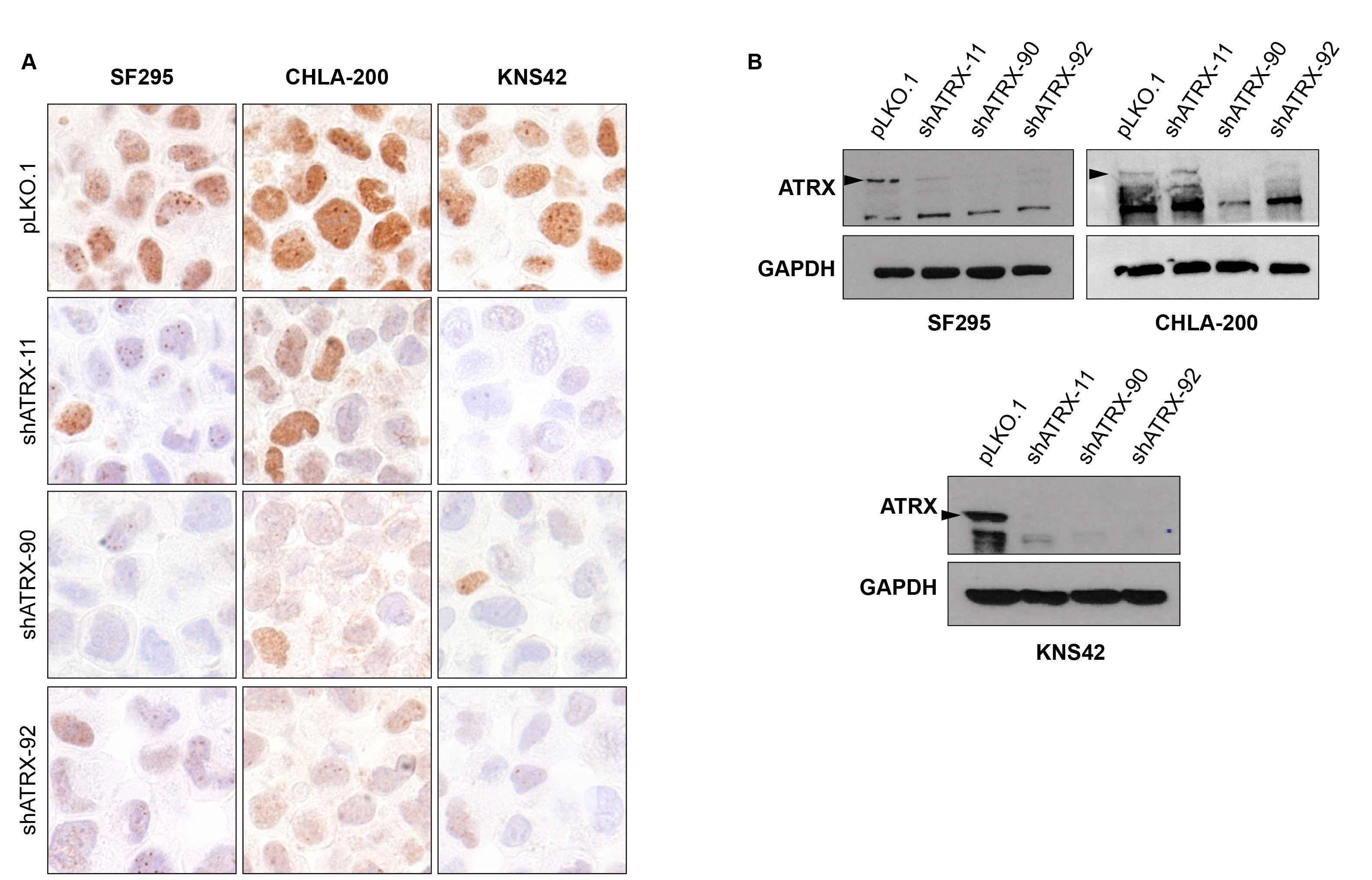

Supplement: S9 Fig — ATRX knockdown in SF295, CHLA-200, and KNS42 was confirmed using (A) immunohistochemistry and (B) immunoblotting against ATRX. Arrowhead indicates band representing full length wild-type ATRX. (TIF) [file pone.0204159.s010.tif]

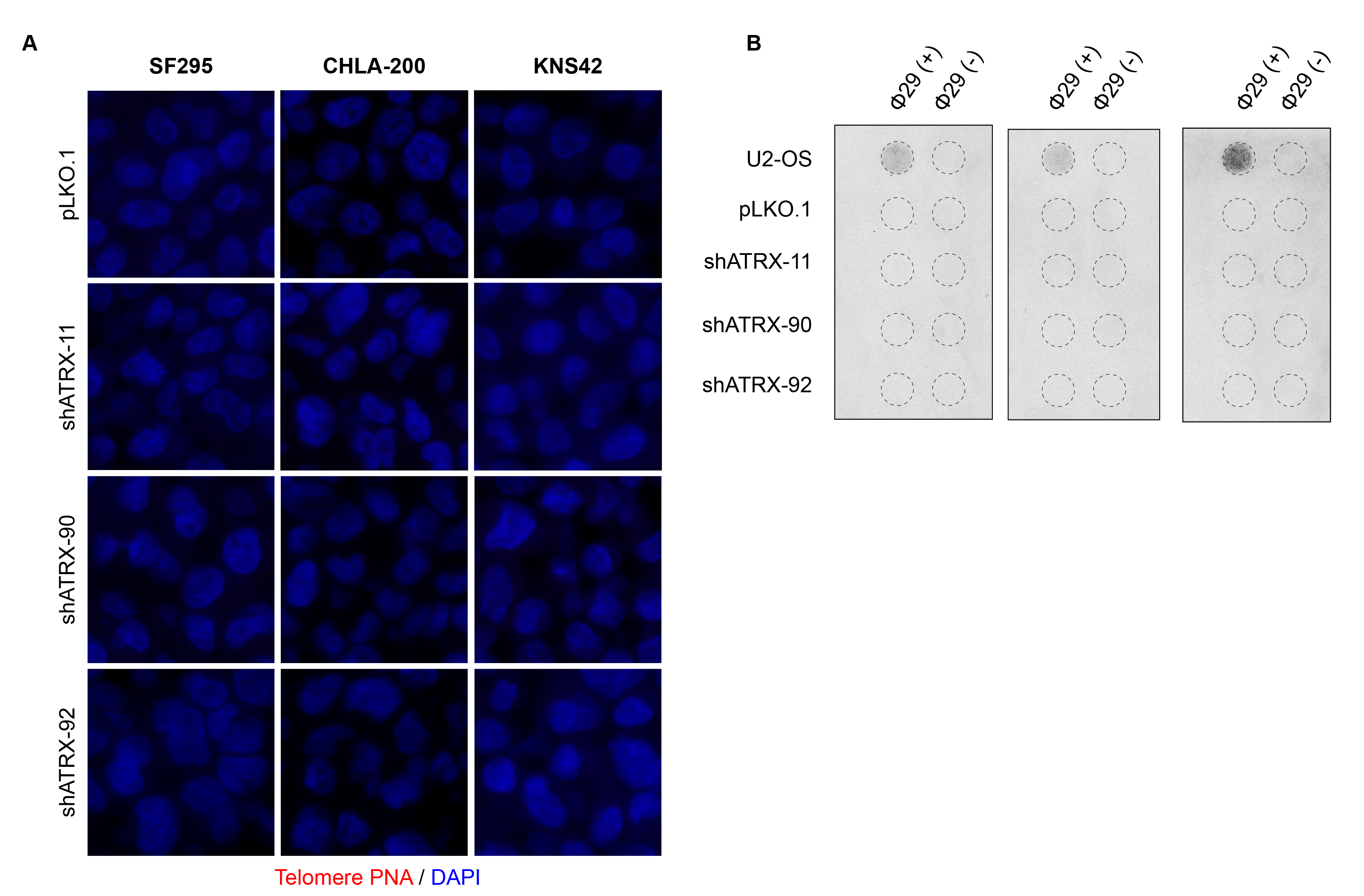

Supplement: S10 Fig — (A) Representative telomere FISH images reveal no telomeric foci formation after ATRX knockdown in SF295, CHLA-200, or KNS42. (B) ATRX knockdown does not induce c-circle formation after ATRX knockdown in SF295, CHLA-200, or KNS42. A lower input of U2-OS DNA (30 ng, compared to 150 ng) was included as a positive control. (TIF) [file pone.0204159.s011.tif]
